# Supplementary material for: Description of a Sarcoptic Mange Outbreak in Alpine Chamois Using an Enhanced Surveillance Approach
Source: Animals (Basel). 2022 Aug 15;12(16):2077. doi: 10.3390/ani12162077 (PMC9405409; doi:10.3390/ani12162077)
Supplement: Supplementary file 1 [file animals-12-02077-s001.zip › Table S1_Mange Strategy.pdf]

Table S1 - Mange management strategy in chamois populations of Trento Province

Type	Description	Prioritary management aims	Management
1	First mange cases in naïve zone	Monitoring	Eventual increase of hunting bag (up to 20% of previous census)
2	Mange cases with no (<15%) population decline	No hunting restrictions	As with no demographic decline
3	Mange cases with moderate (15-30%) population decline	No hunting restrictions	Hunting bag < 10% of previous census
4	Mange cases with evident (30-40%) population decline	Spare resistent animals; limit disturbance on infected animals	Hunting bag < 5% of previous census
5	Mange cases with evident (>40%) population decline	Spare resistent animals; limit disturbance on infected animals	No hunting
6	Naïve zone bordering affected areas	Monitoring	Eventual increase of hunting bag (up to 20% of previous census)
7	Post epidemic situation (applicable for no more than 3 years)	Promote demographic recovery (≥50% of pre-epidemic status).	Hunting bag is defined according to the effective demography recovery. Hunting must return to normality following these steps:<br>• 50-70% of pre-epidemic stocks: hunting bag ≤5% of census data<br>• 70-80% of pre-epidemic stocks: hunting bag ≤10% of census data<br>• ≥80%: ordinary hunting bag
