# Supplementary material for: Description of a Sarcoptic Mange Outbreak in Alpine Chamois Using an Enhanced Surveillance Approach
Source: Animals (Basel). 2022 Aug 15;12(16):2077. doi: 10.3390/ani12162077 (PMC9405409; doi:10.3390/ani12162077)
Supplement: Supplementary file 1 [file animals-12-02077-s001.zip › Table S2_Summary of Mange strategy in mange zones.pdf]

Table S2 - Summary of Mange strategy in mange zones 2006-2020

**Mange zone**	**2006**	**2007**	**2008**	**2009**	**2010**	**2011**	**2012**	**2013**	**2014**	**2015**	**2016**	**2017**	**2018**	**2019**	**2020**
PAN	Type 3	Type 3	Type 3	Type 3	Type 3	Type 4	Type 4	Type 5	Type 5	Type 7	Type 7	Type 7	OM	OM	OM
ROL	Type 6	Type 2	Type 5	Type 5	Type 5	Type 5	Type 5	Type 5	Type 5	Type 5	Type 5	Type 5	Type 5	Type 5	Type 5
LIT	OM	OM	OM	OM	OM	OM	Type 2	Type 3	Type 3	Type 4	Type 4	Type 3	Type 3	Type 3	Type 3
STE	OM	OM	OM	OM	OM	OM	Type 2	Type 3	Type 3	Type 3	Type 3	Type 4	Type 4	Type 4	Type 4
CAU	Type 1	Type 1	Type 1	Type 3	Type 3	Type 4	Type 4	Type 5	Type 5	Type 7	Type 7	Type 7	OM	OM	OM
LSO	OM	OM	OM	OM	OM	OM	OM	Type 2	Type 2	Type 3	Type 3	Type 7	Type 7	Type 7	OM
SCA	Type 2	Type 2	Type 2	Type 2	Type 2	Type 2	Type 2	Type 5	Type 5	Type 5	Type 5	Type 5	Type 5	Type 7	Type 7
TOT	OM	OM	OM	OM	OM	OM	OM	OM	Type 1	Type 2	Type 2	Type 3	Type 4	Type 5	Type 6
PAL	Type 1	Type 1	Type 1	Type 3	Type 3	Type 3	Type 4	Type 4	Type 3	Type 3	Type 3	Type 3	Type 7	Type 7	Type 7
OLT	OM	OM	OM	OM	OM	OM	OM	OM	Type 4	Type 4	Type 2	Type 2	Type 7	Type 7	Type 7
VFS[1]						OM	OM	OM	OM	OM	Type 2	Type 5	Type 5	Type 5	Type 5
VFM[1]						OM	OM	OM	OM	OM	OM	OM	Type 4	Type 5	Type 5

OM - Ordinary management

[1]Vette Feltrine management unit was split into VFS and VMS in 2011
