# Supplementary figures and images for: Description of a Sarcoptic Mange Outbreak in Alpine Chamois Using an Enhanced Surveillance Approach
Source: Animals (Basel). 2022 Aug 15;12(16):2077. doi: 10.3390/ani12162077 (PMC9405409; doi:10.3390/ani12162077)

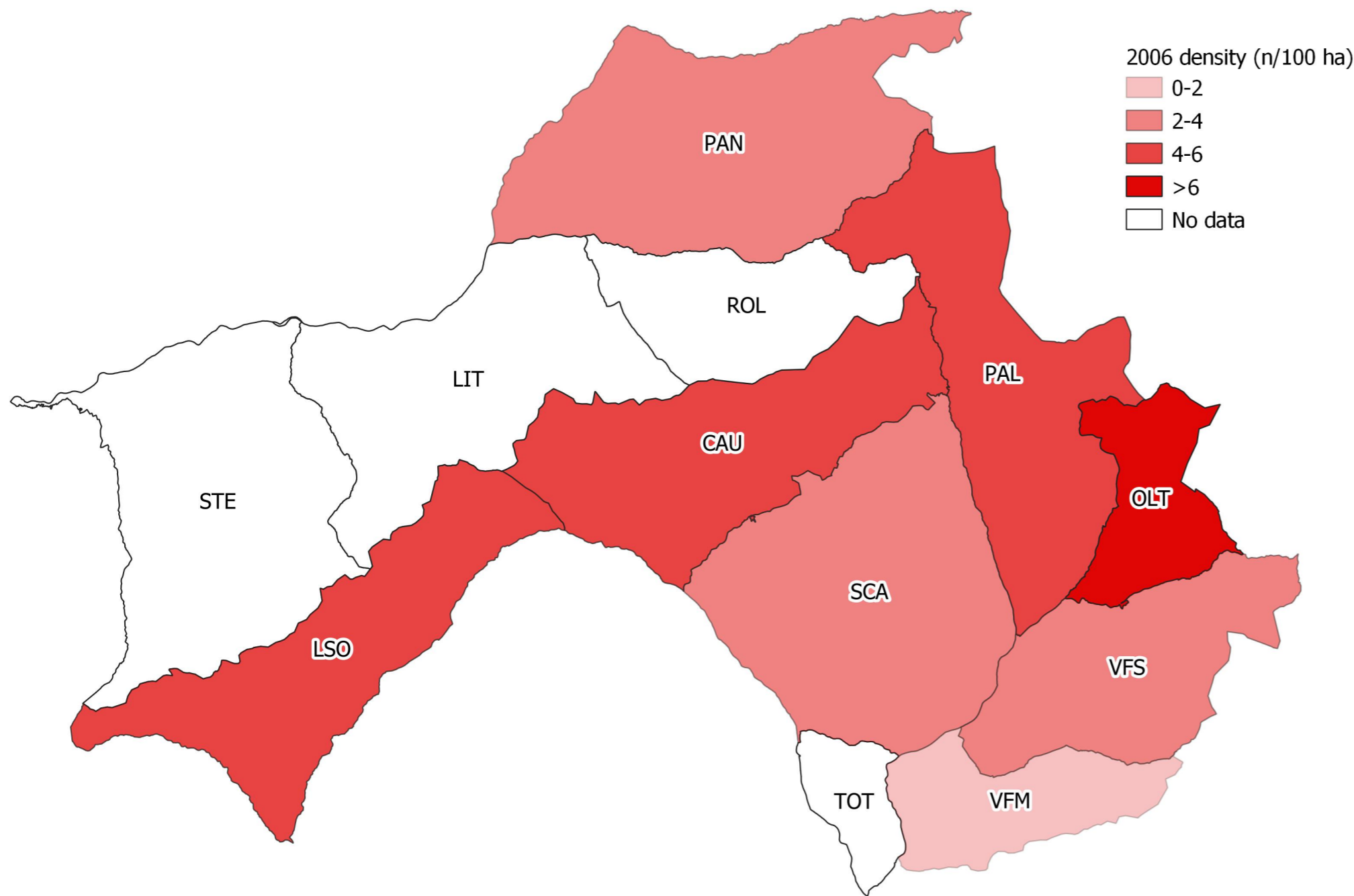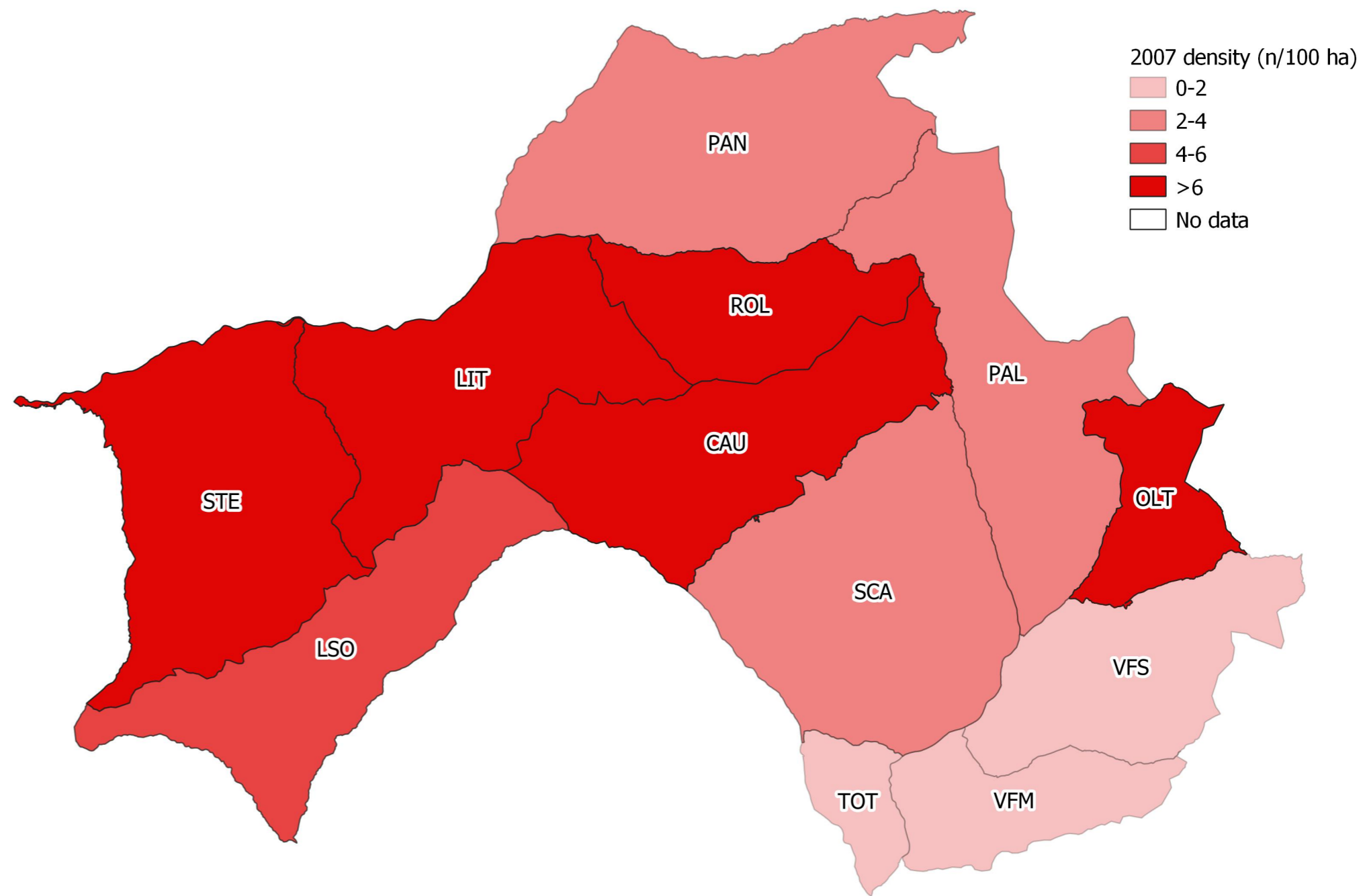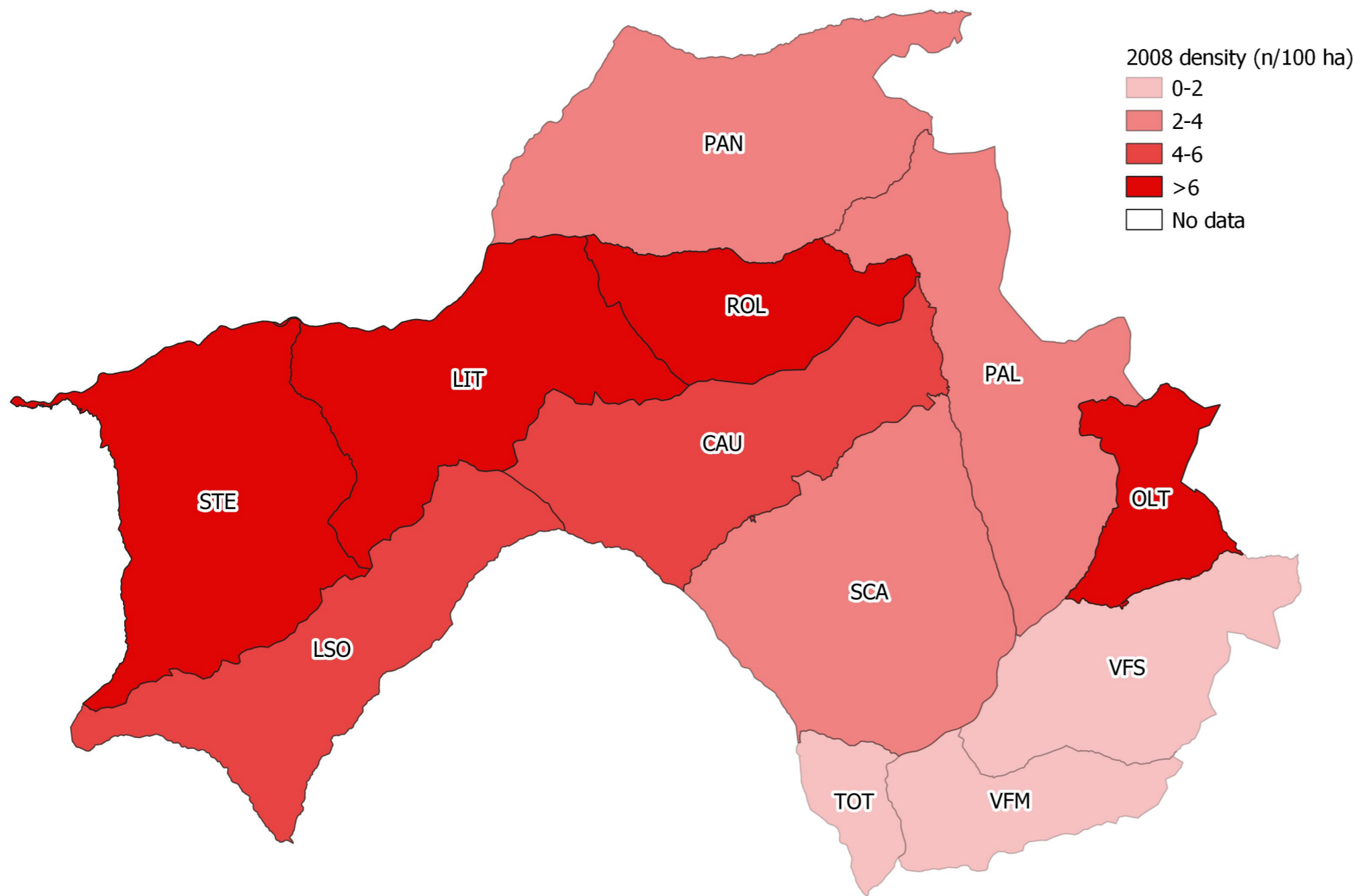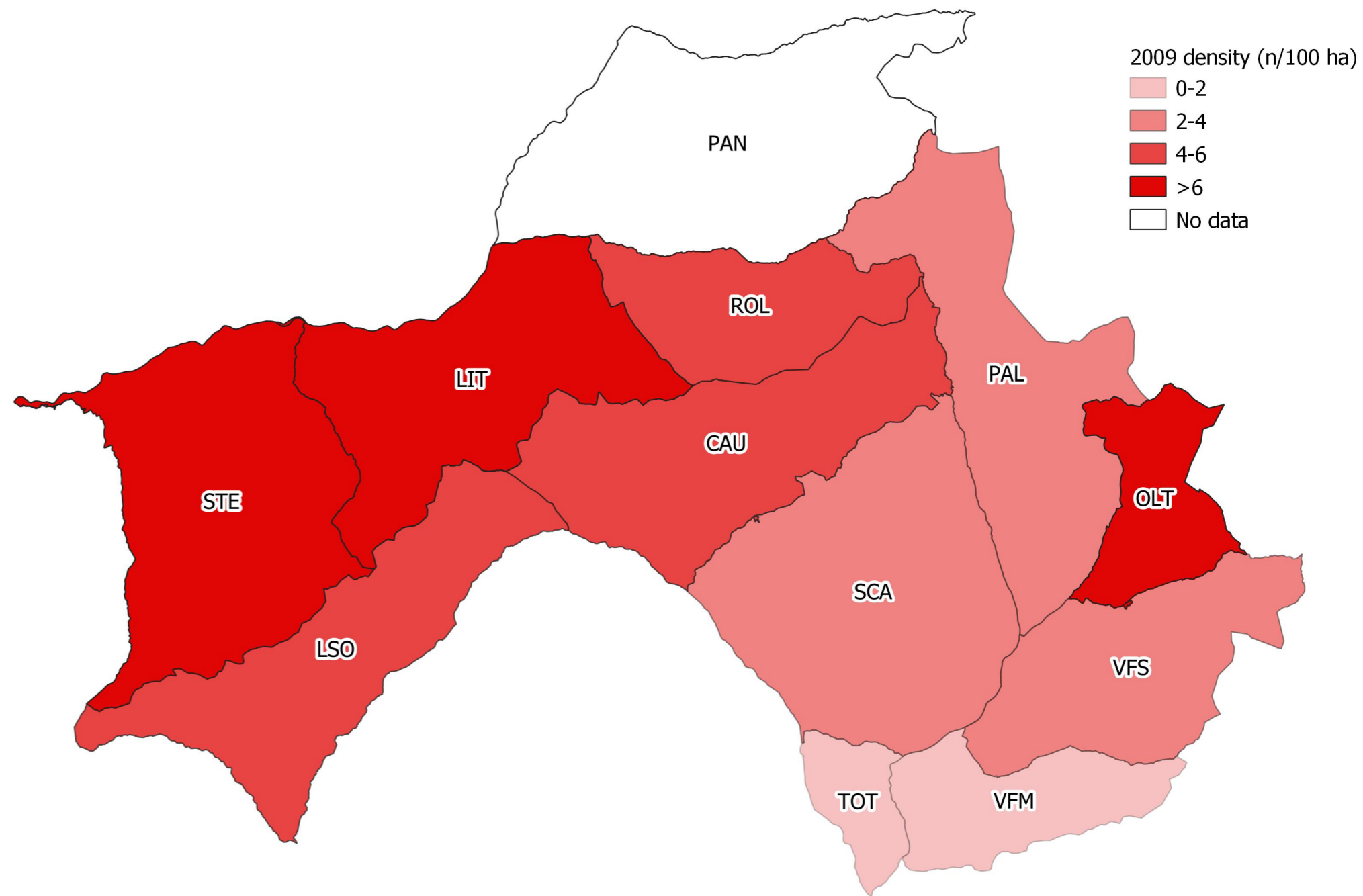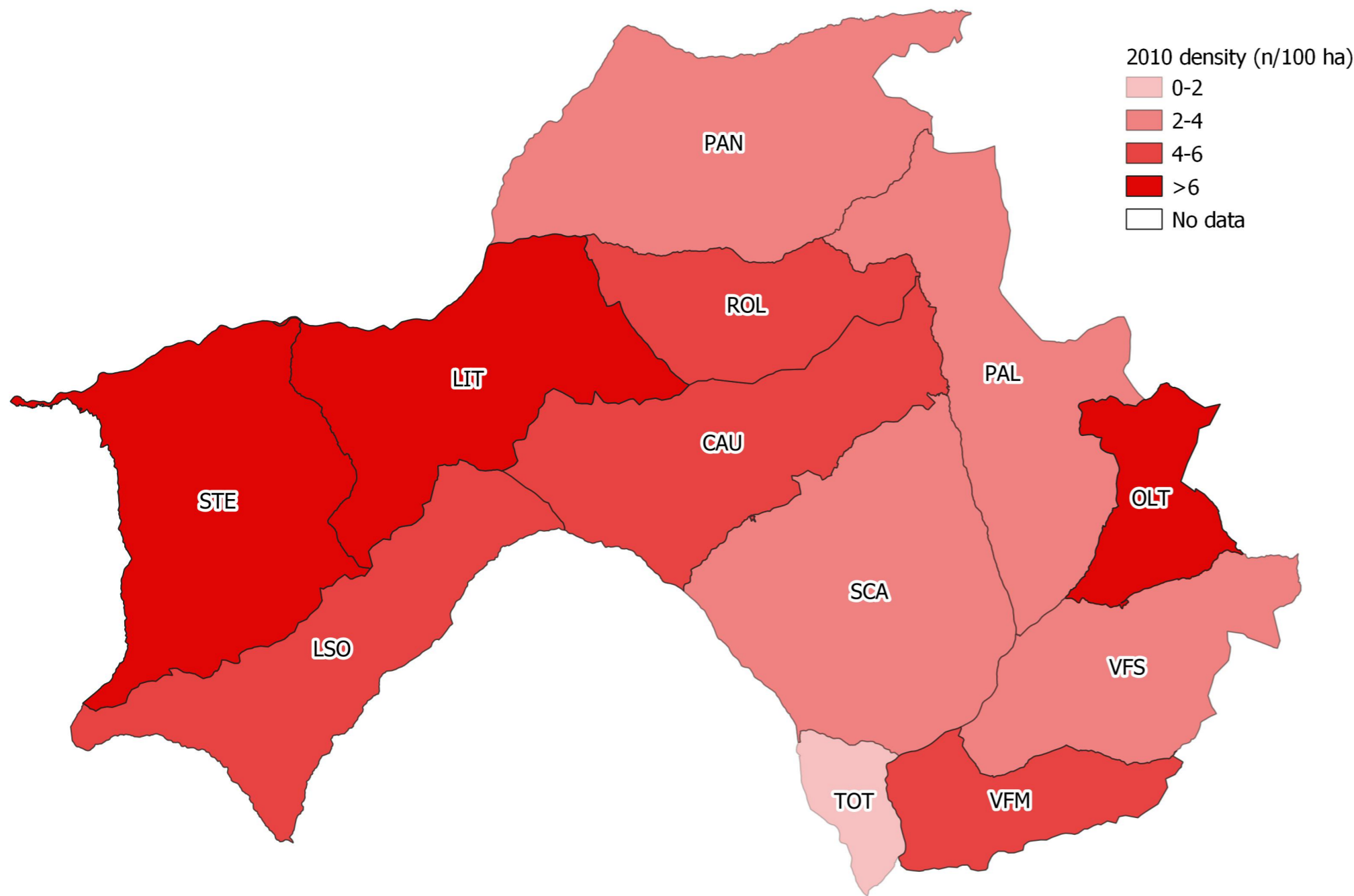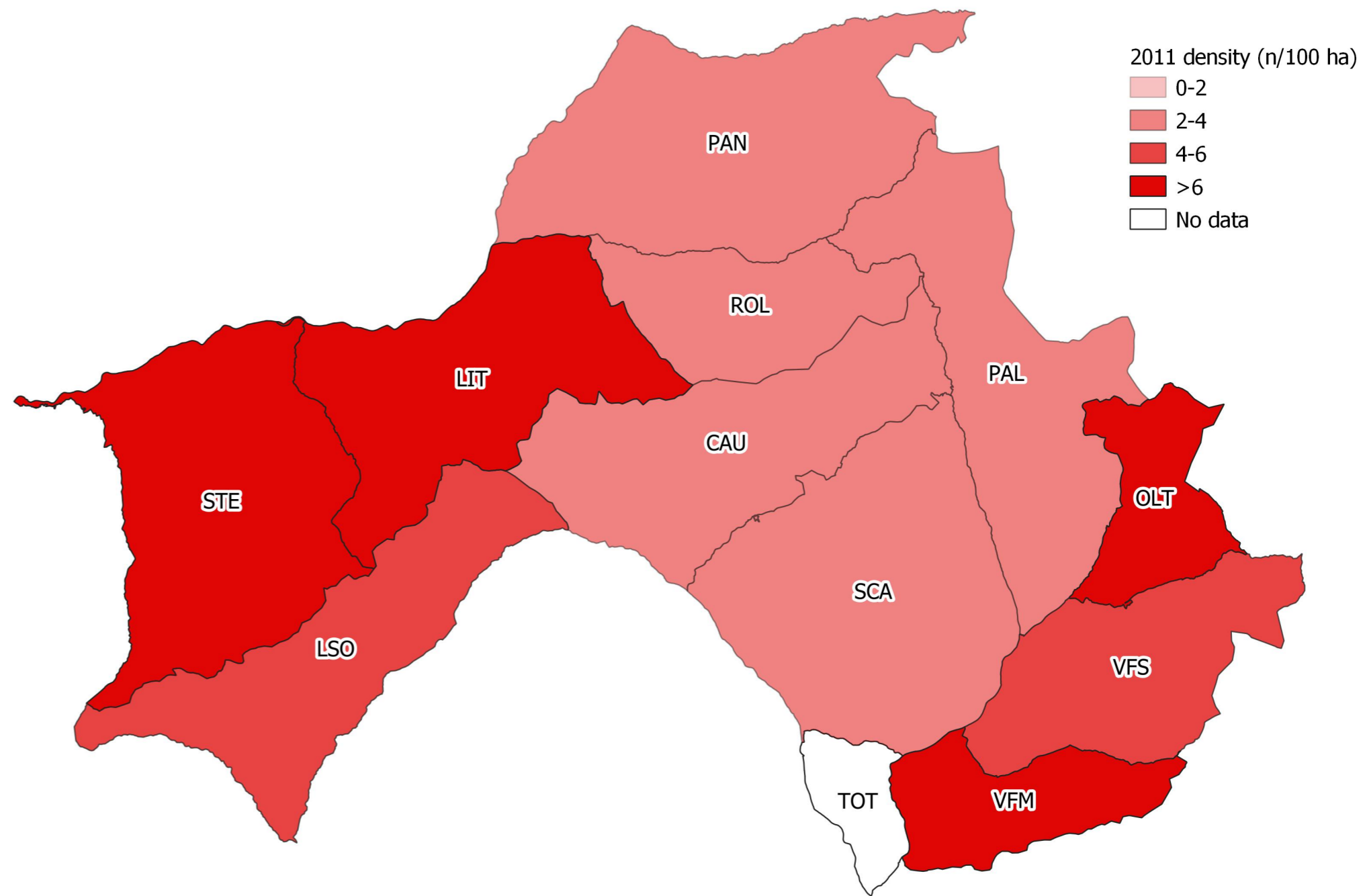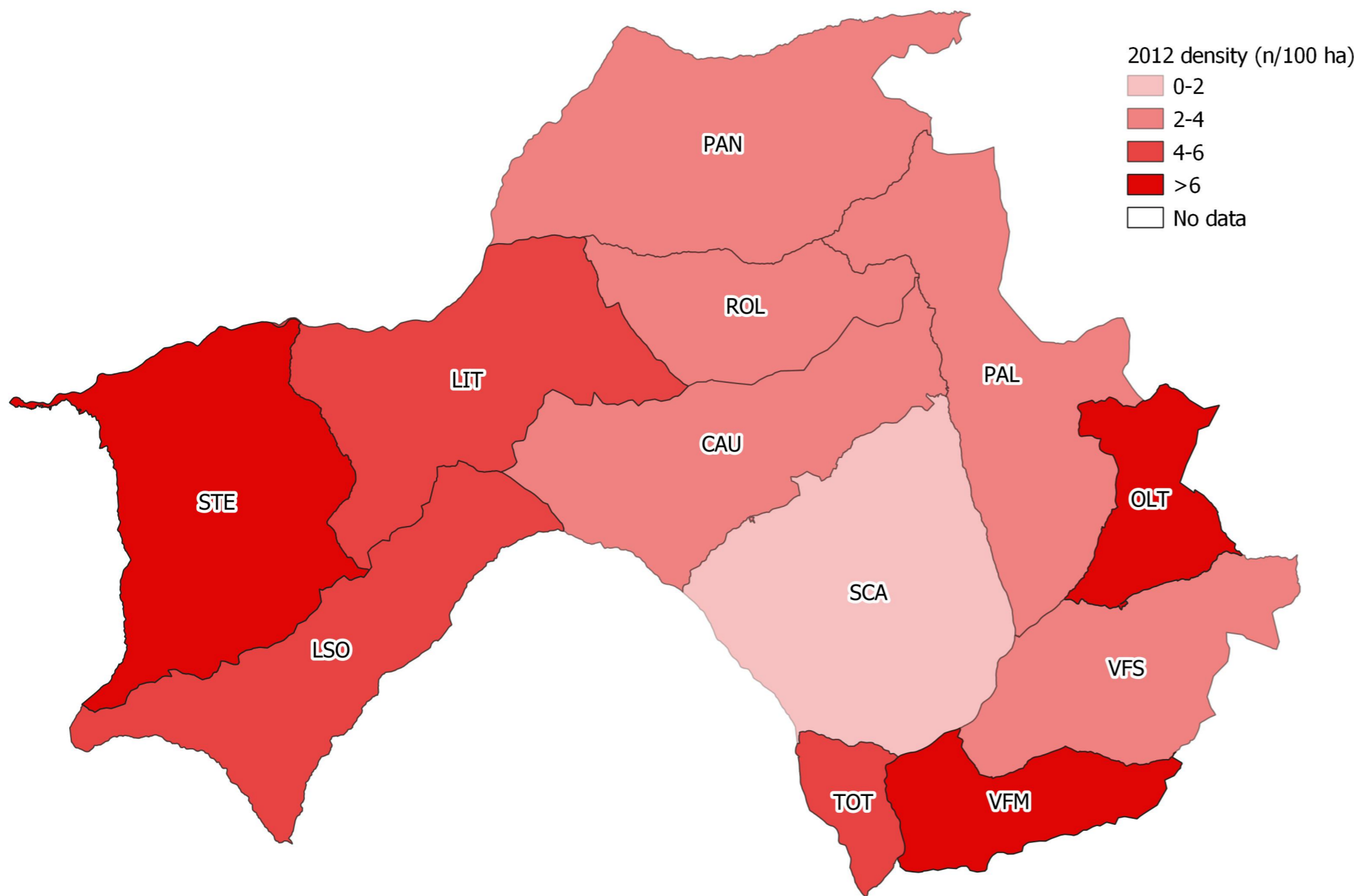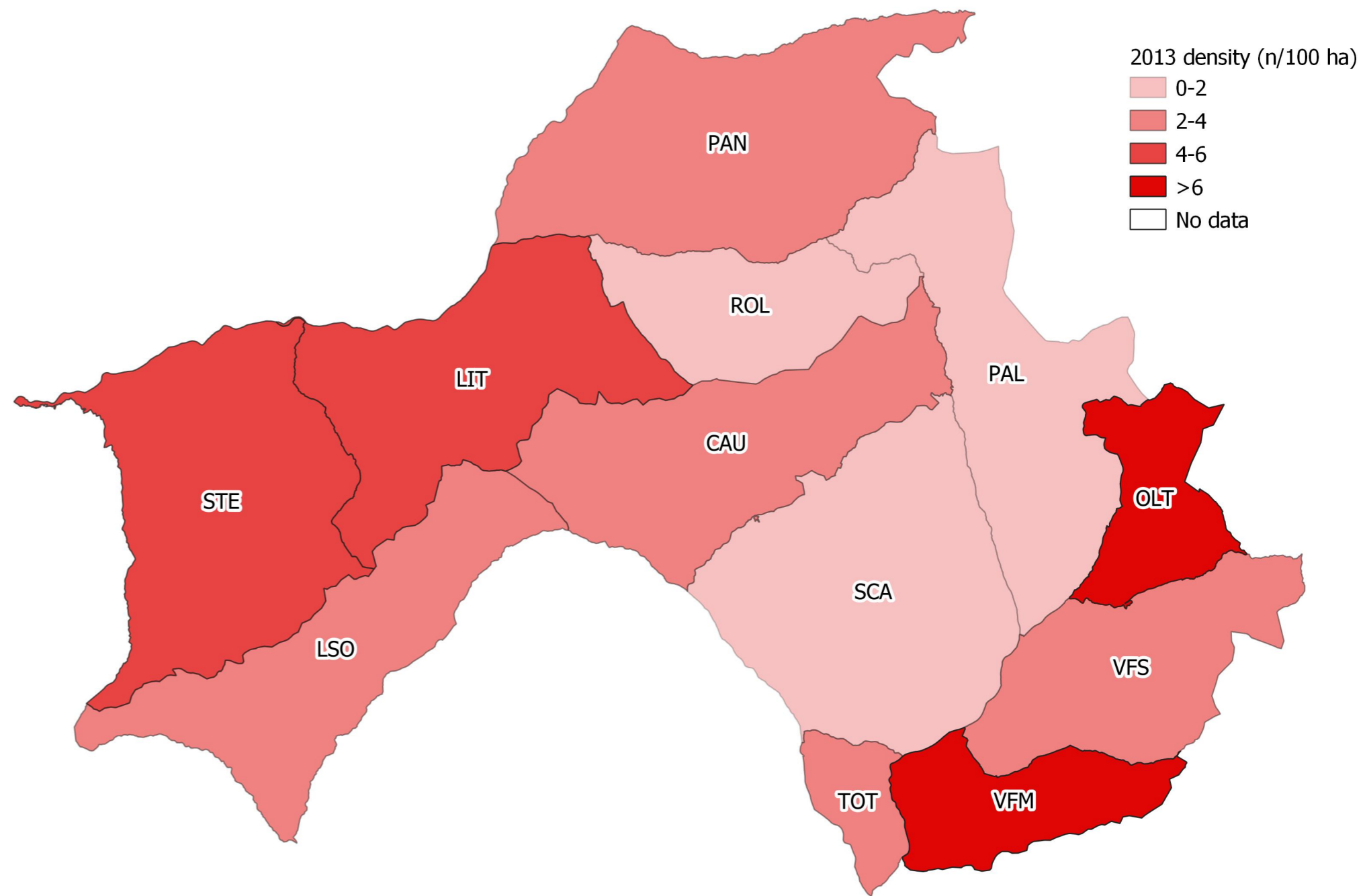

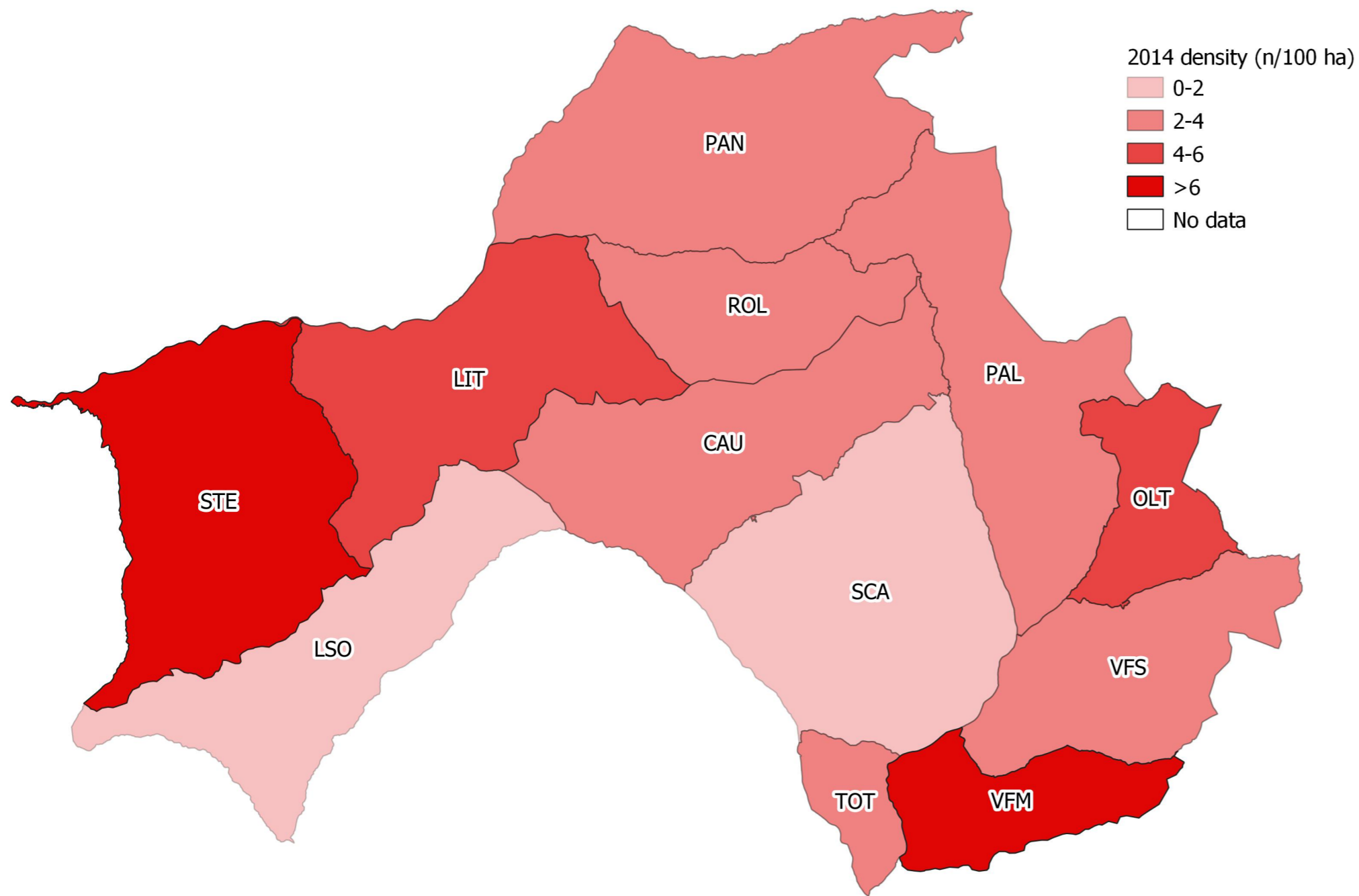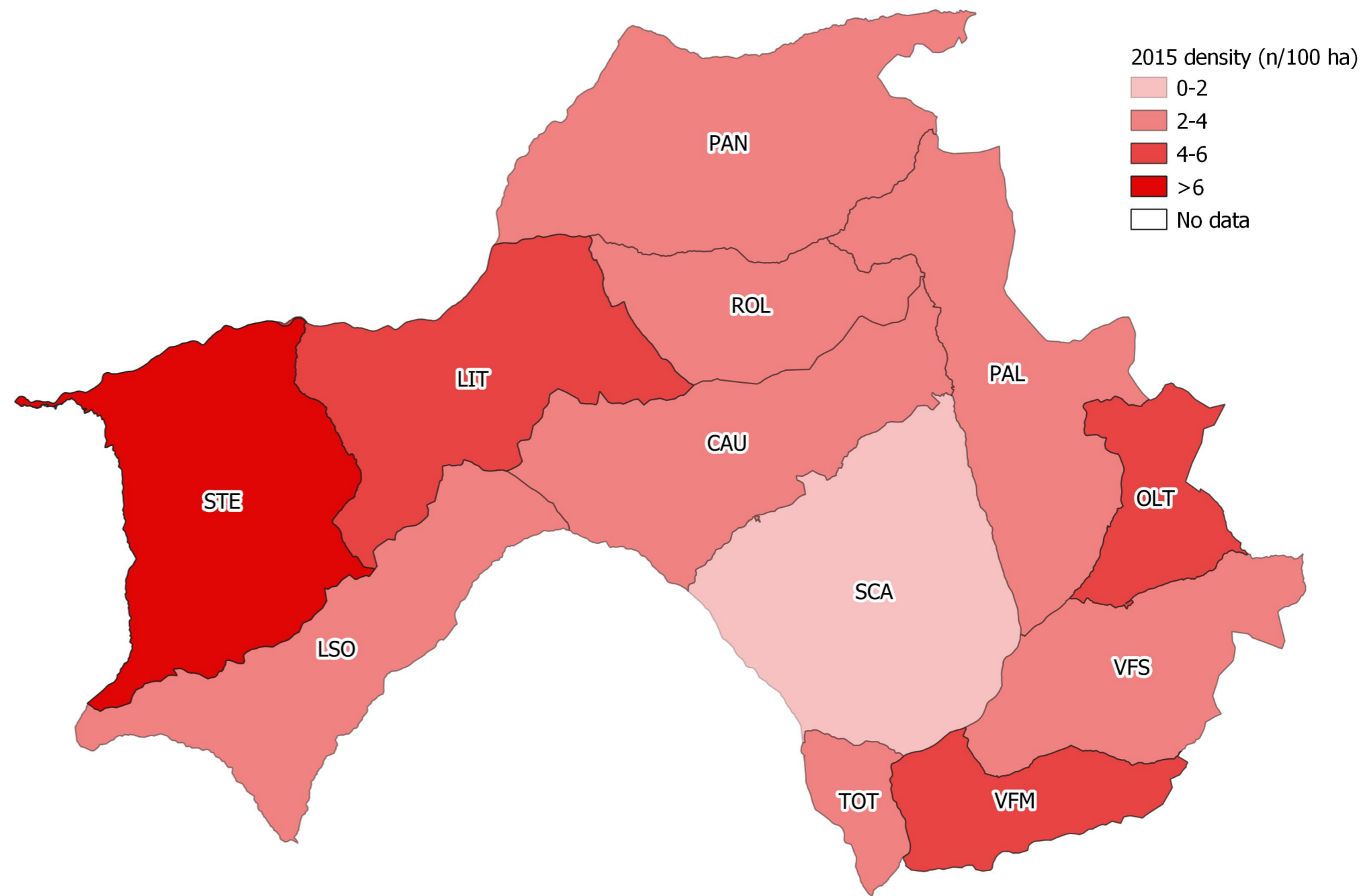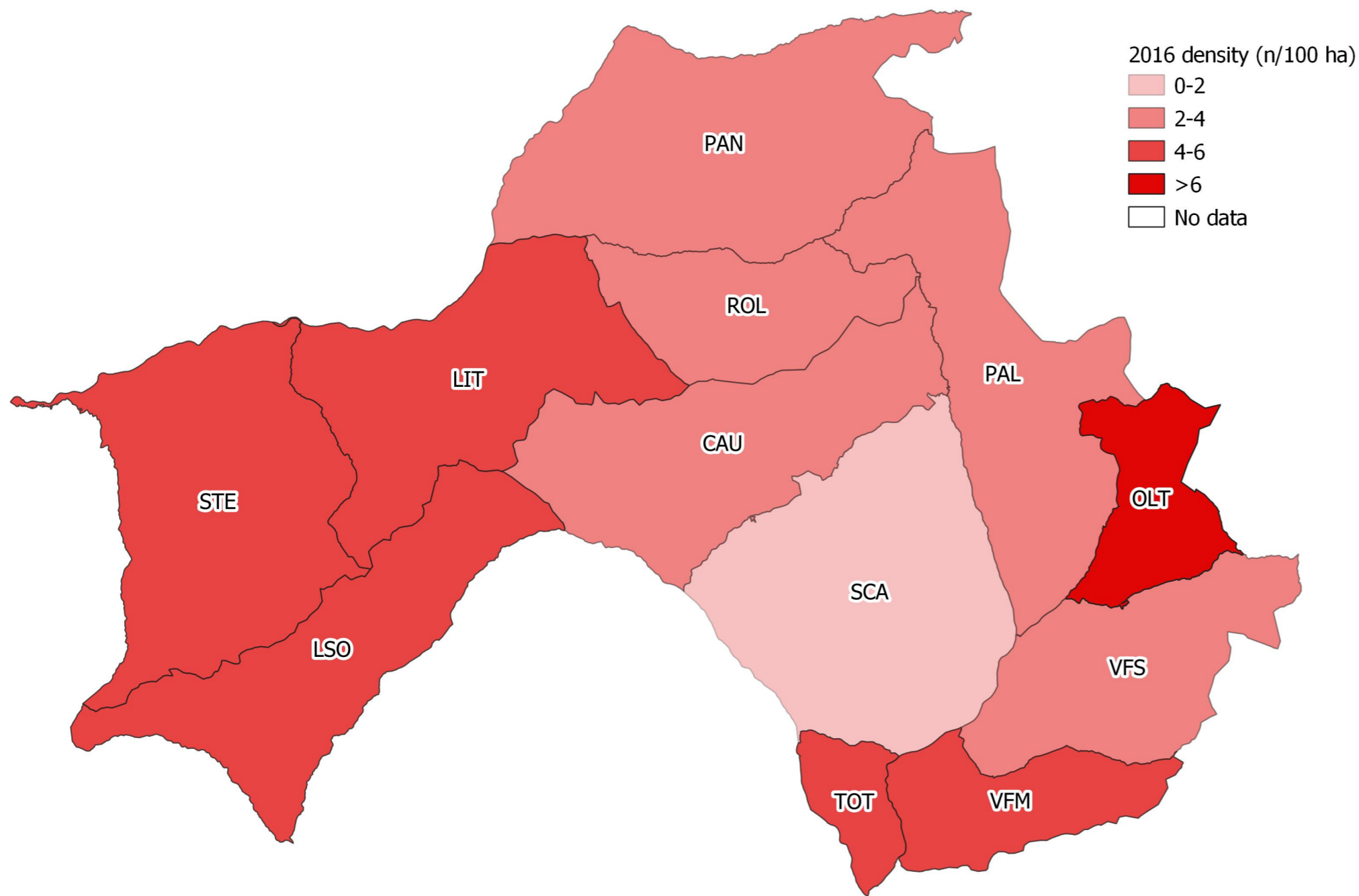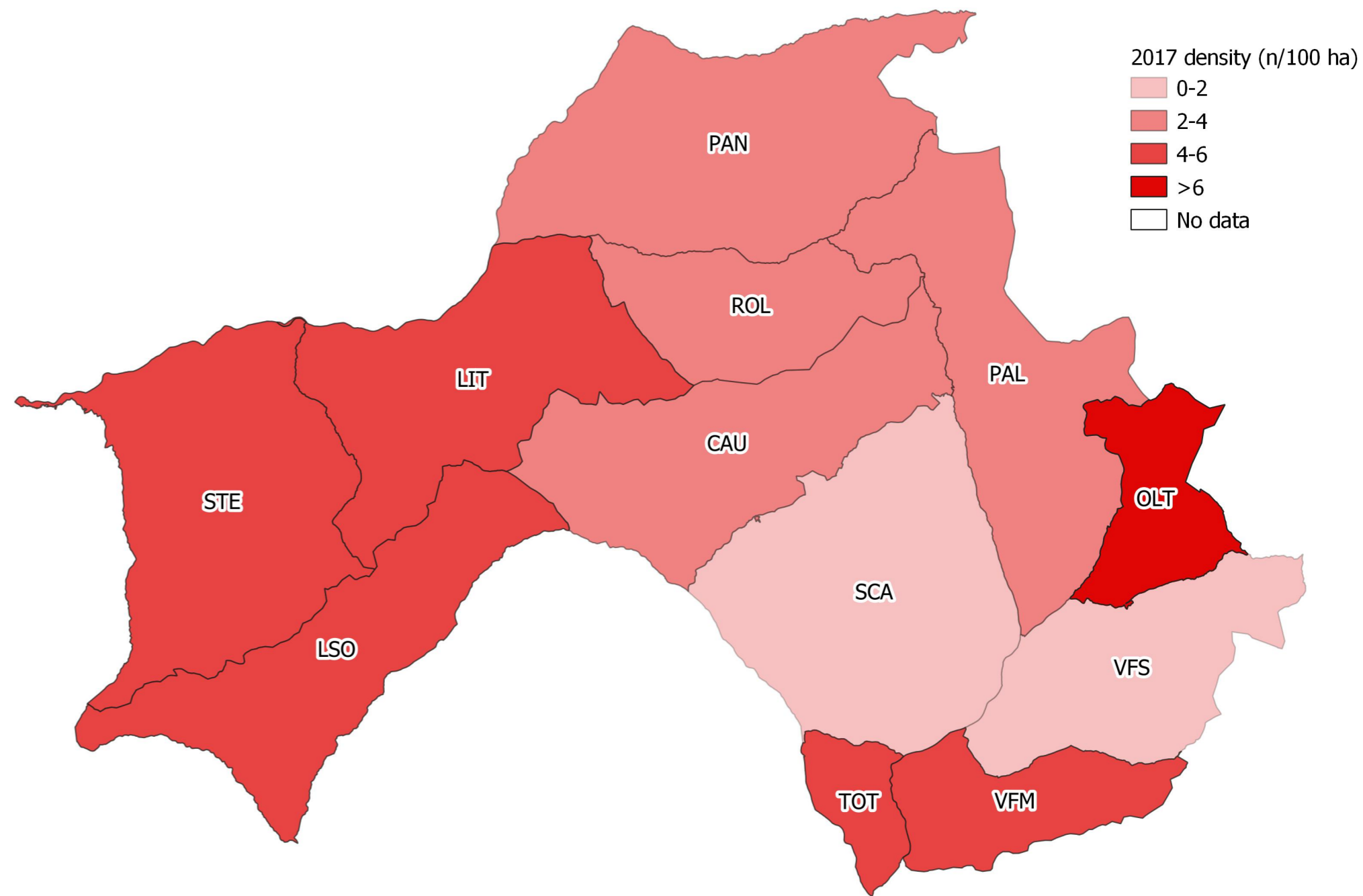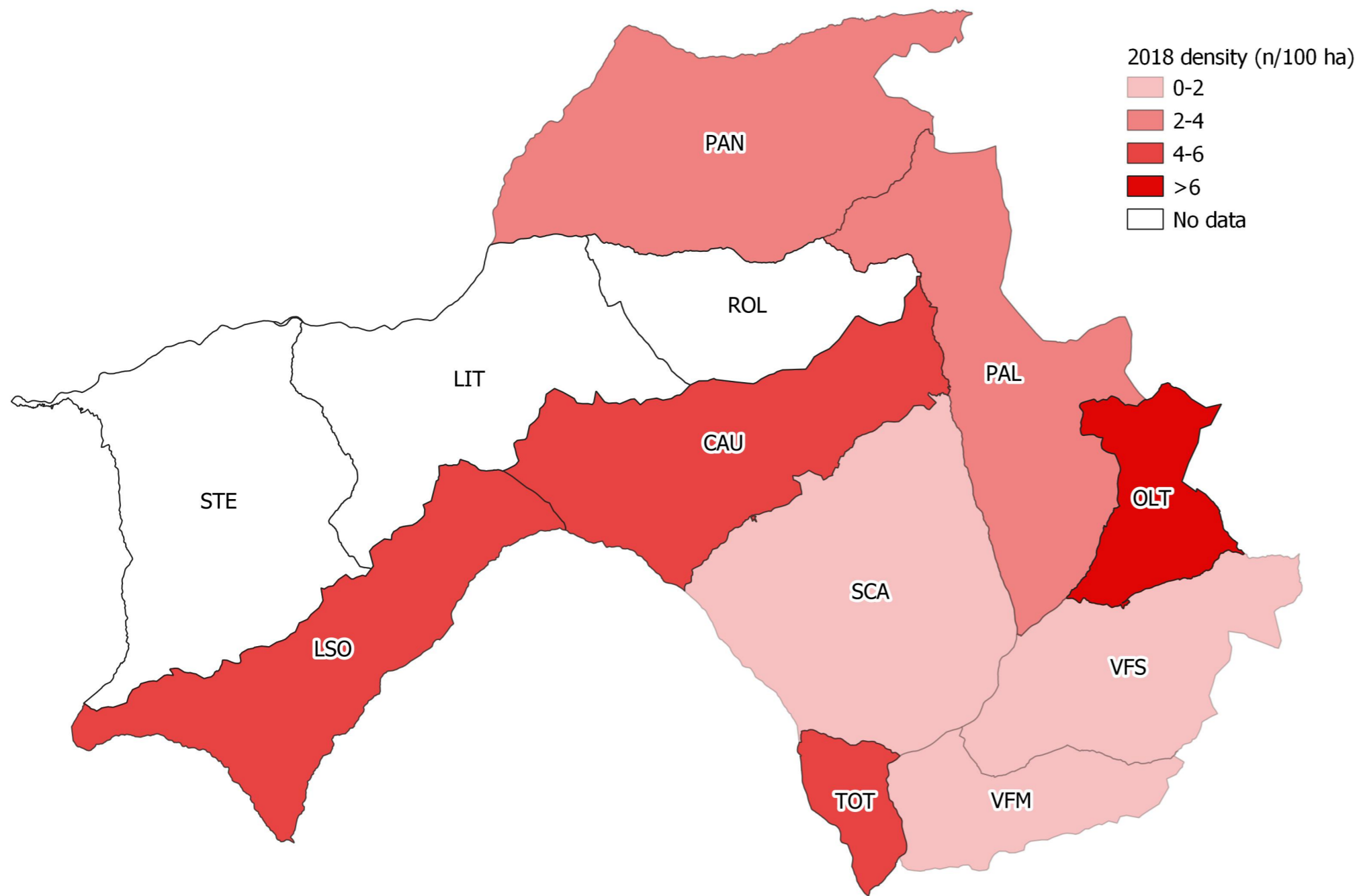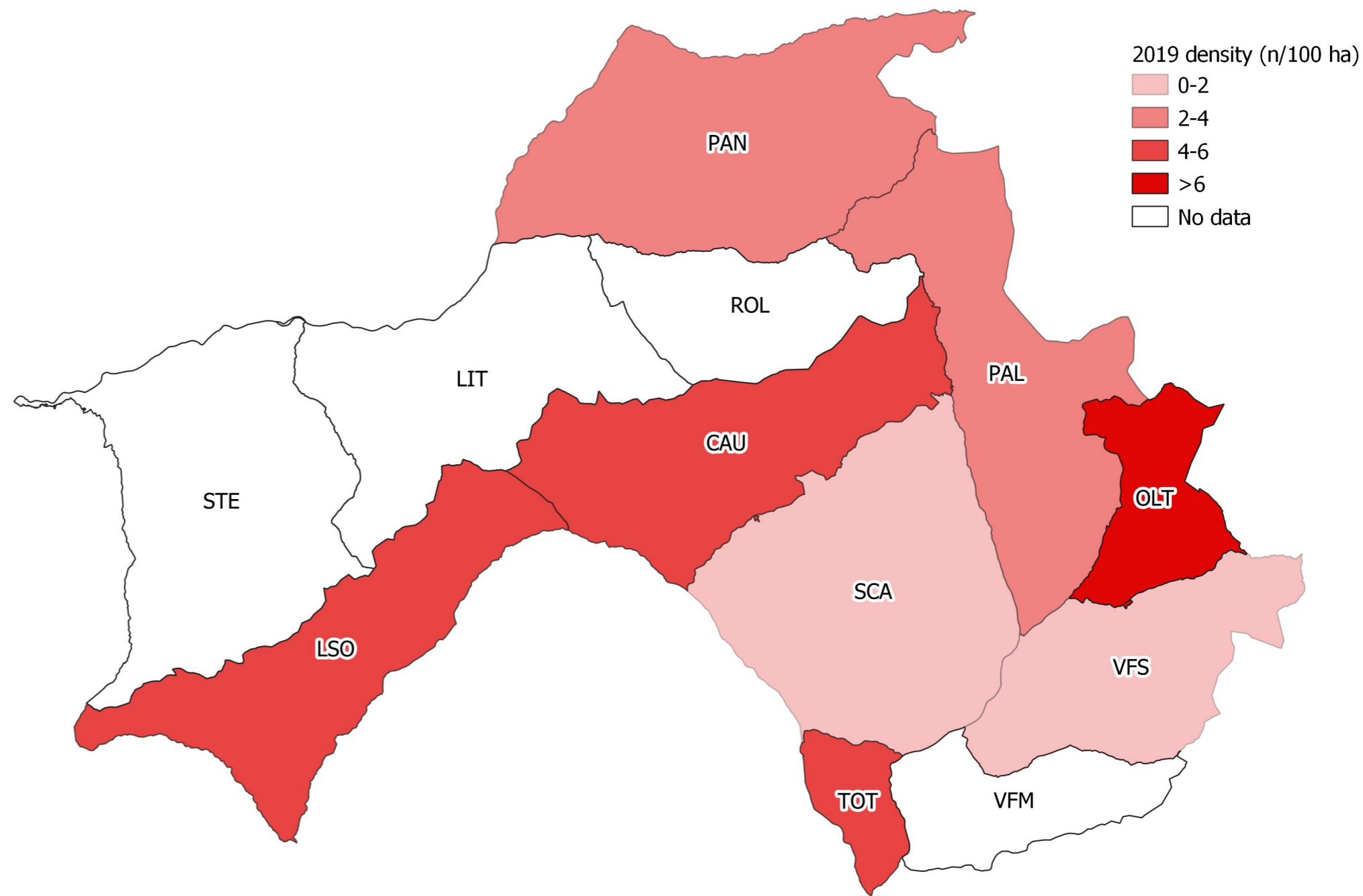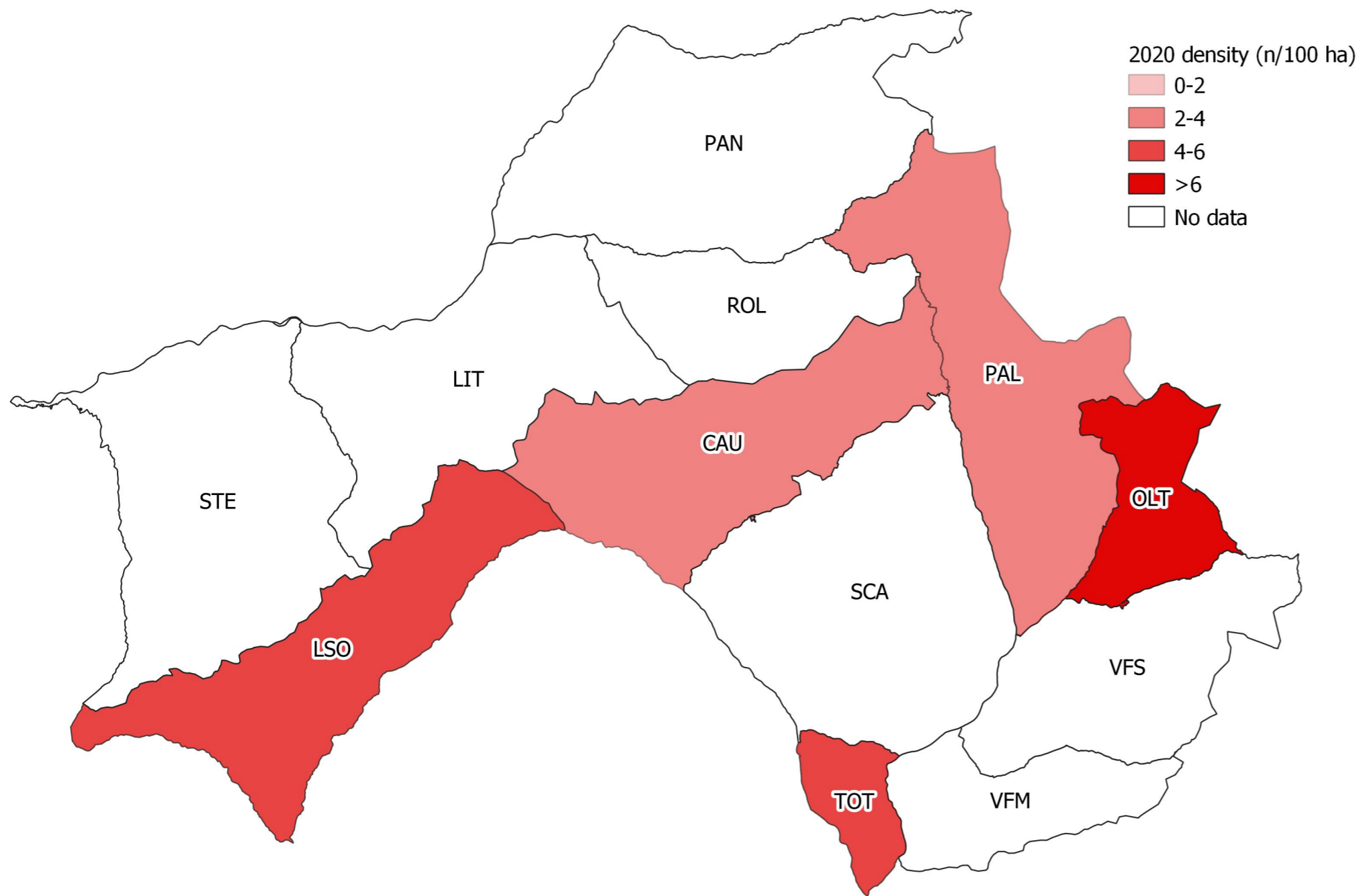

Supplement: Supplementary file 1 [file animals-12-02077-s001.zip › Figure S1_Density maps.pdf]

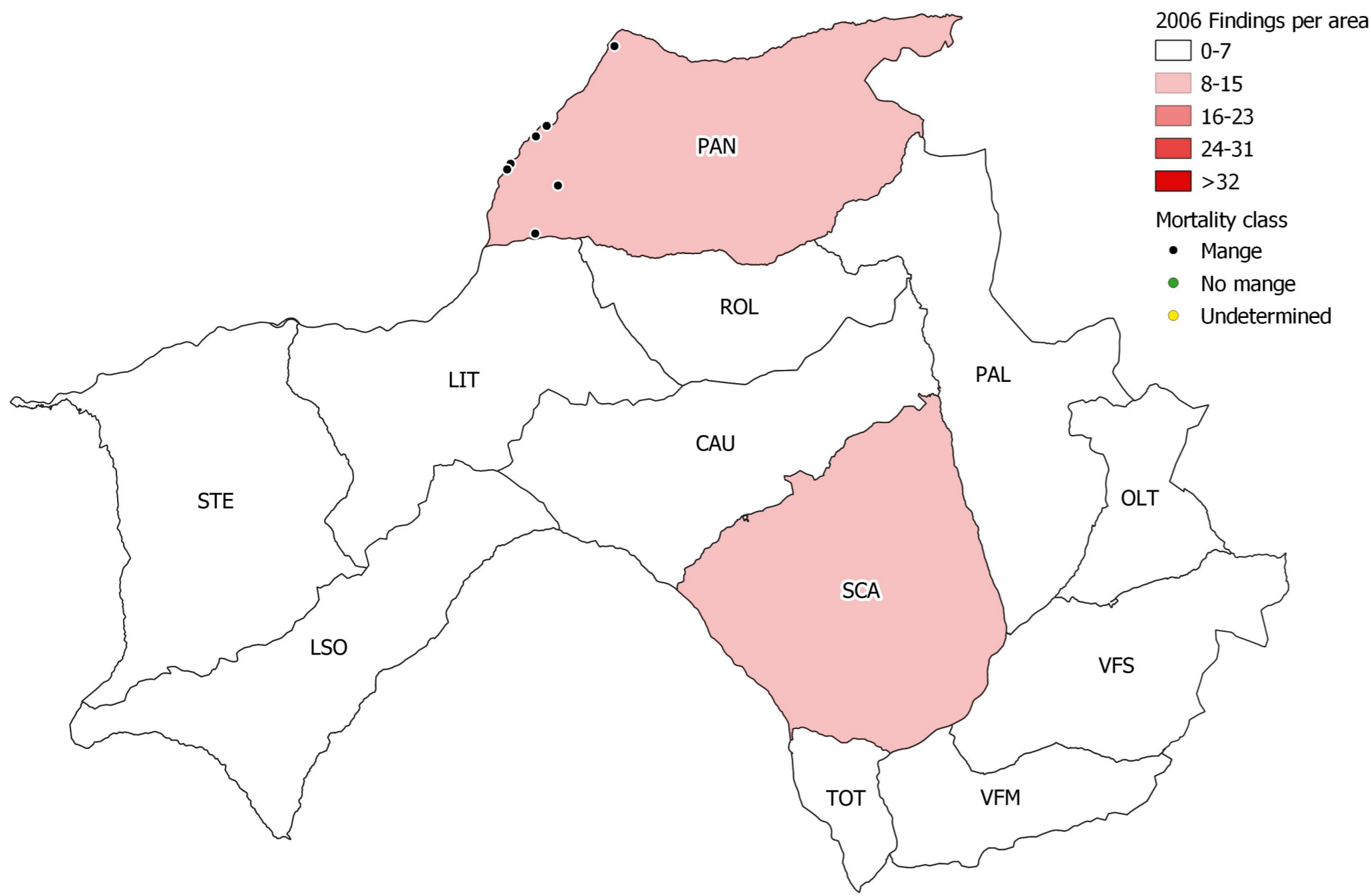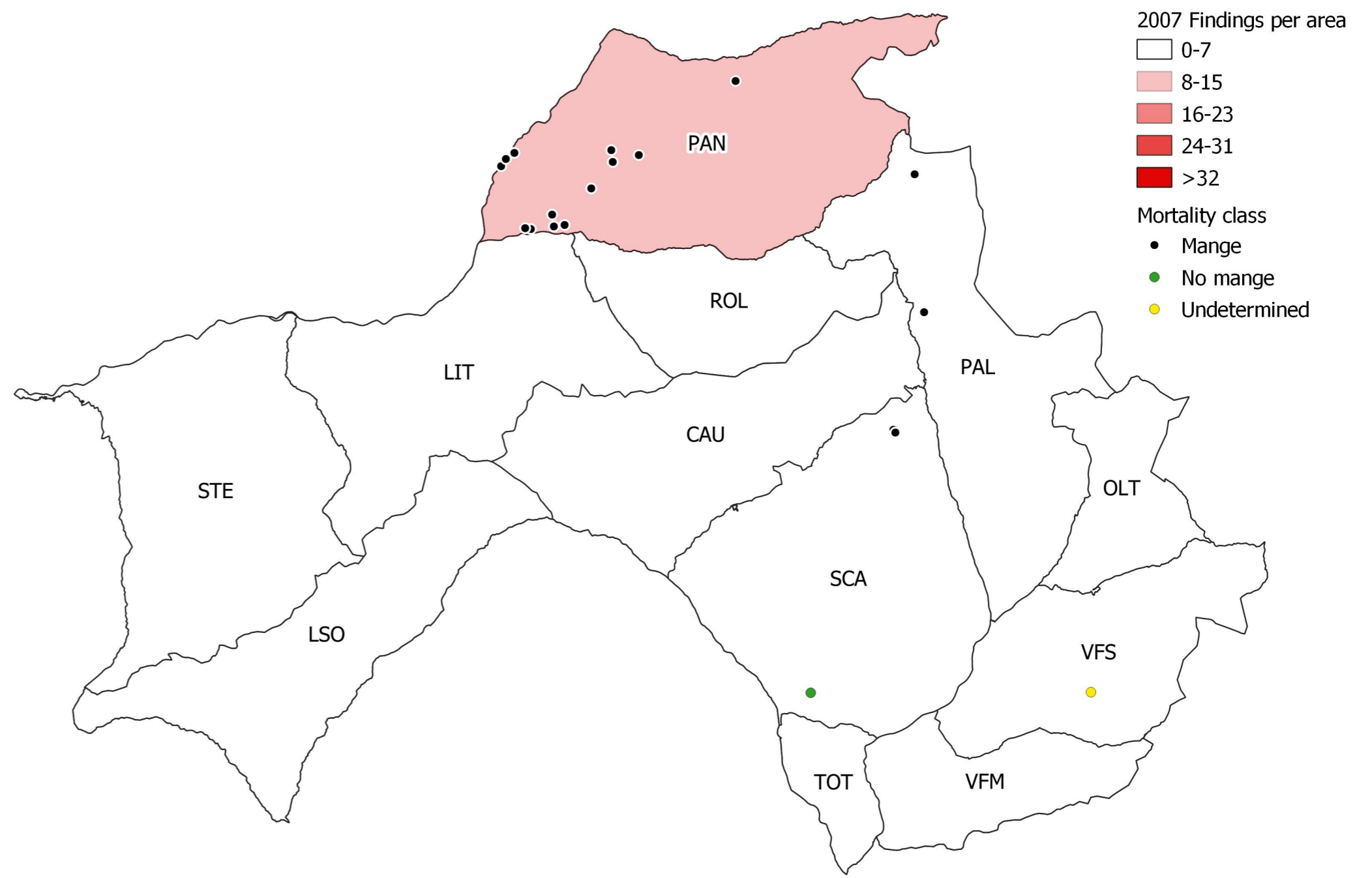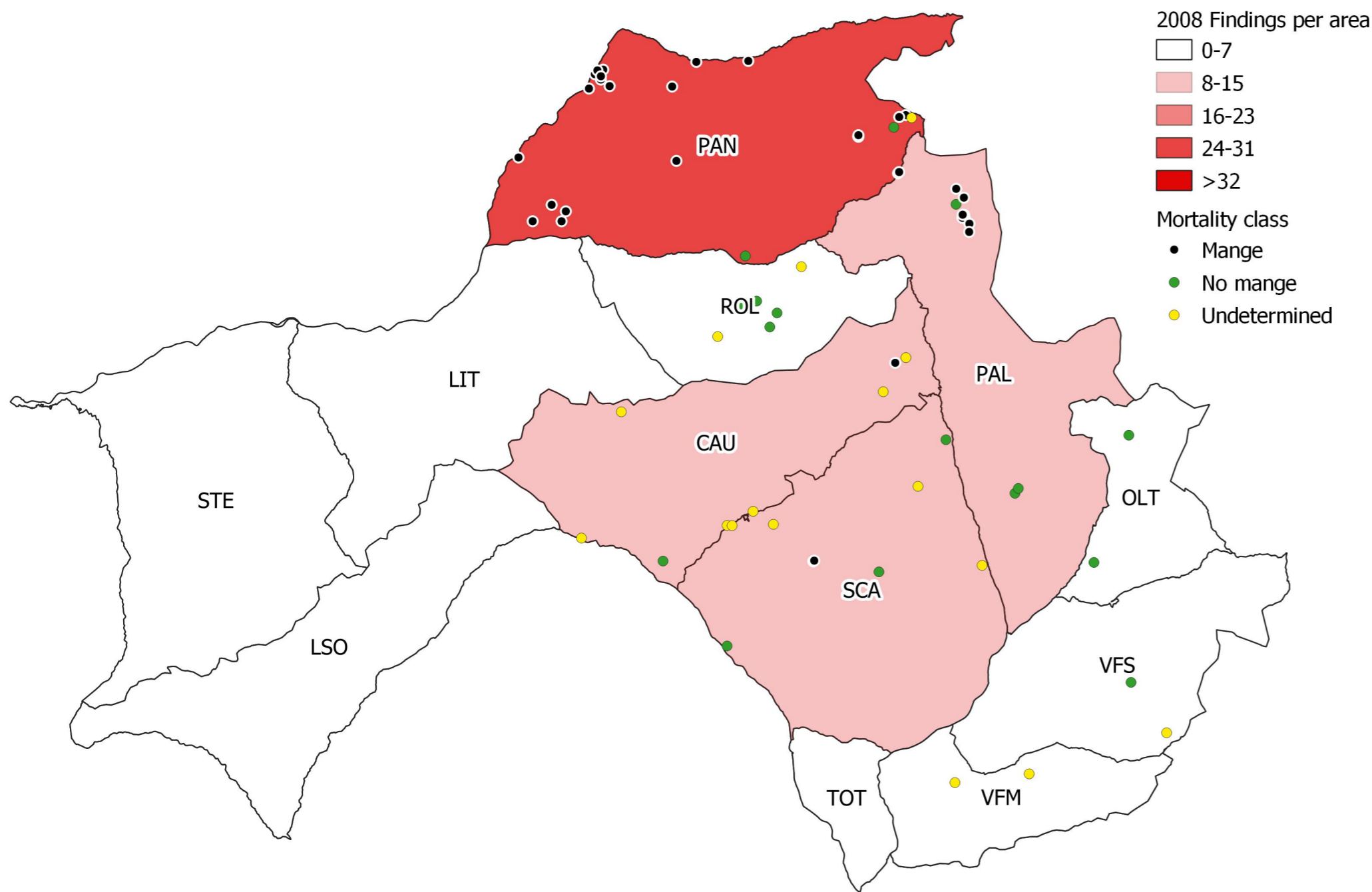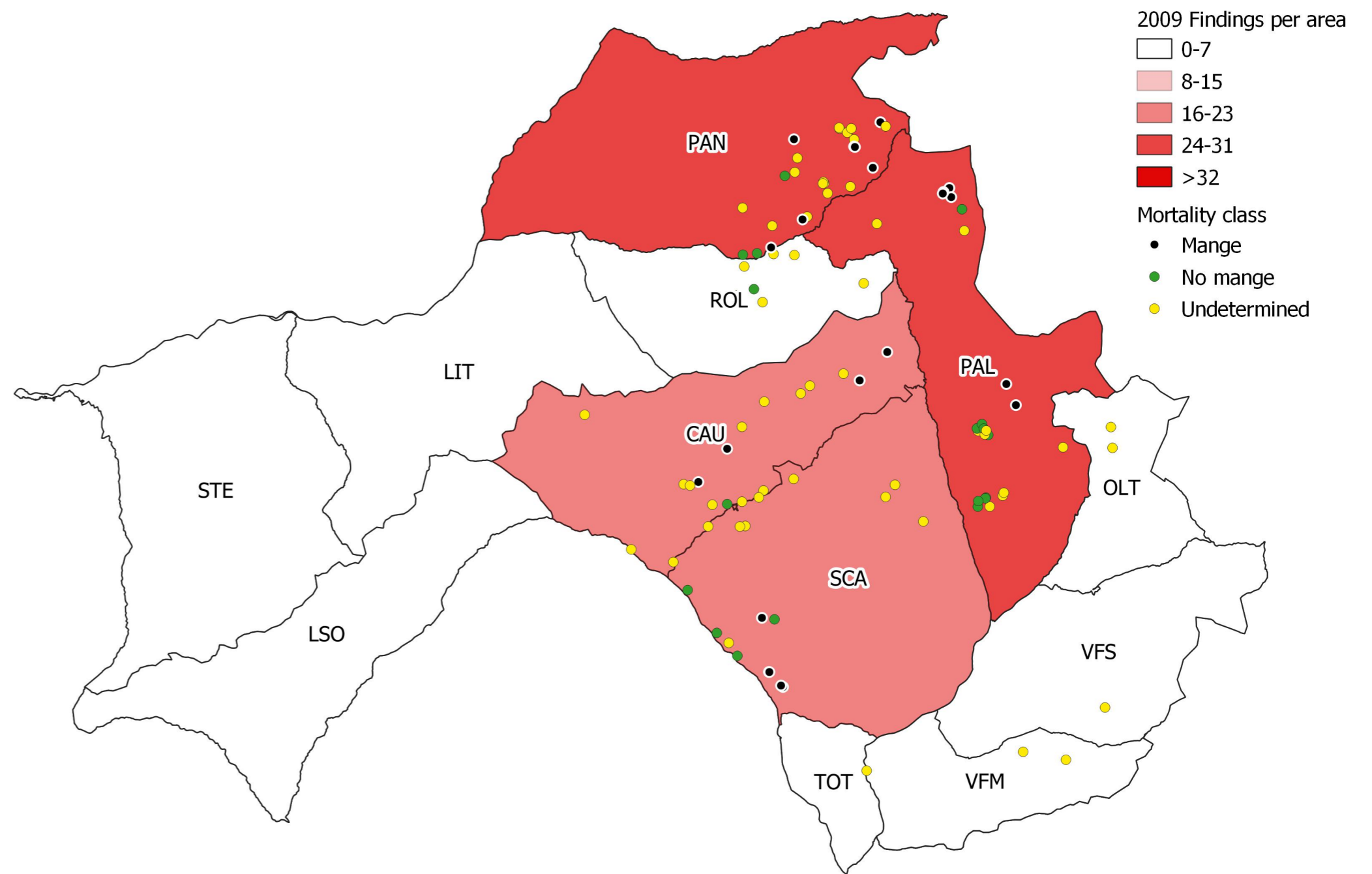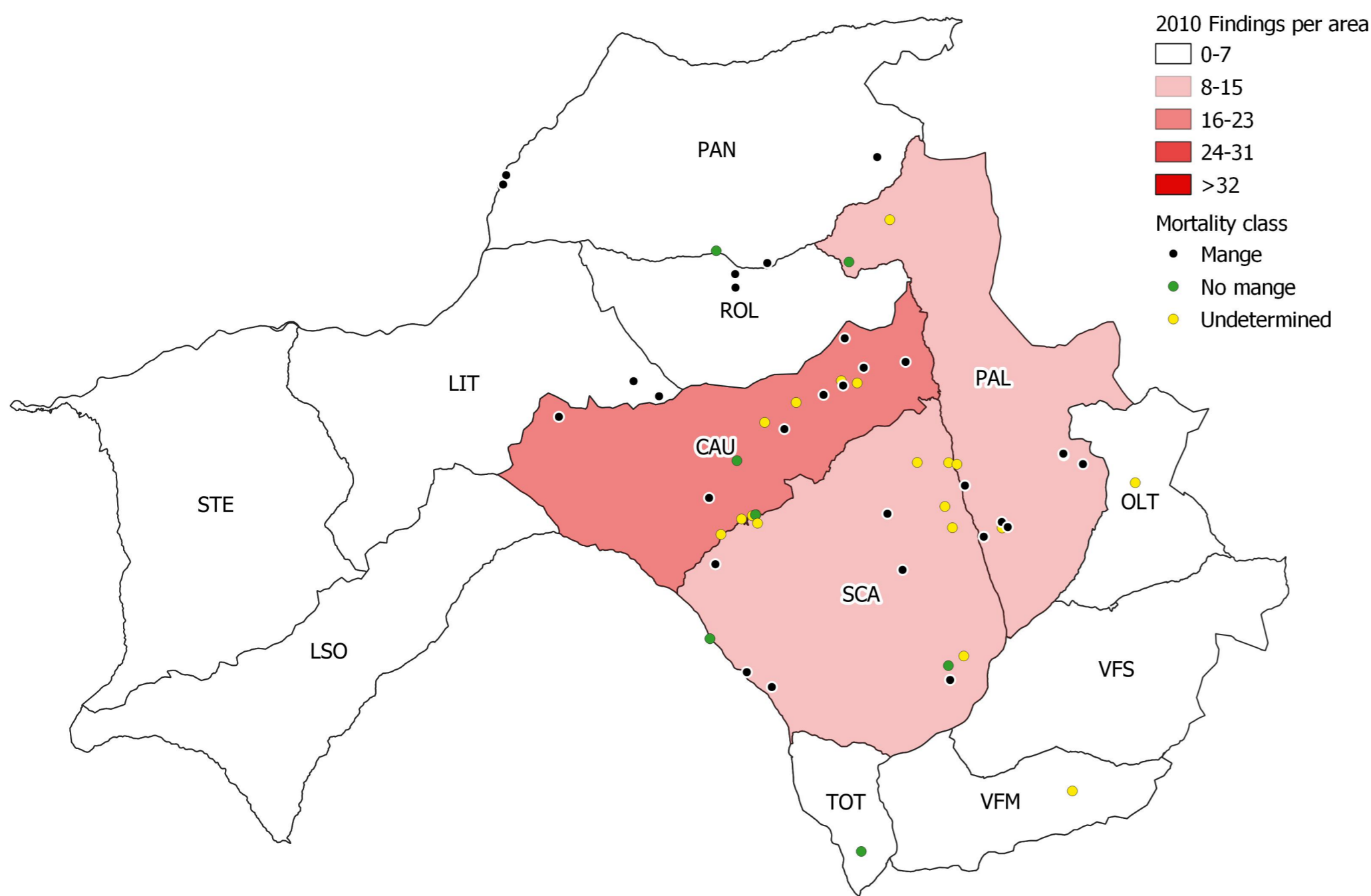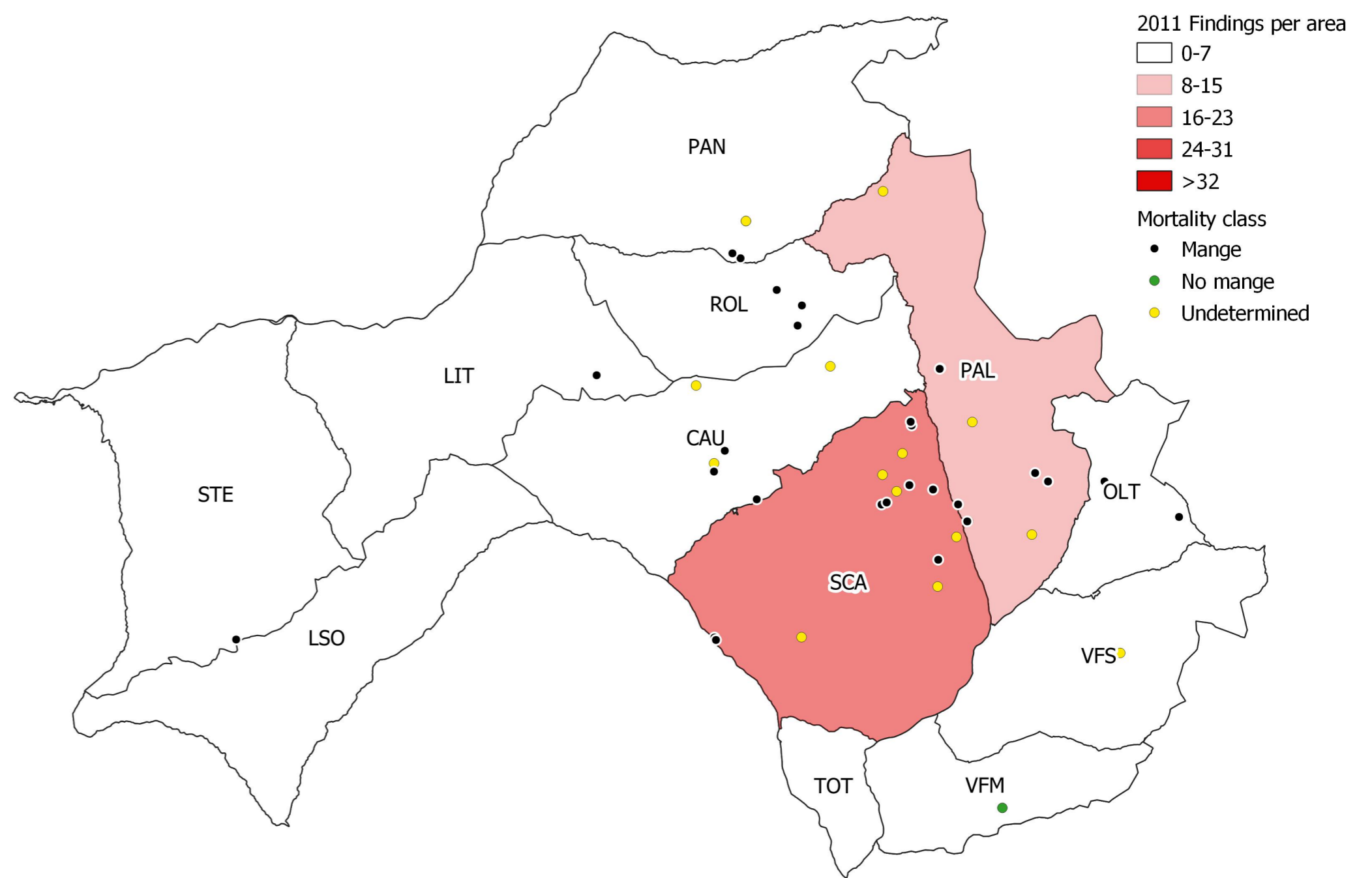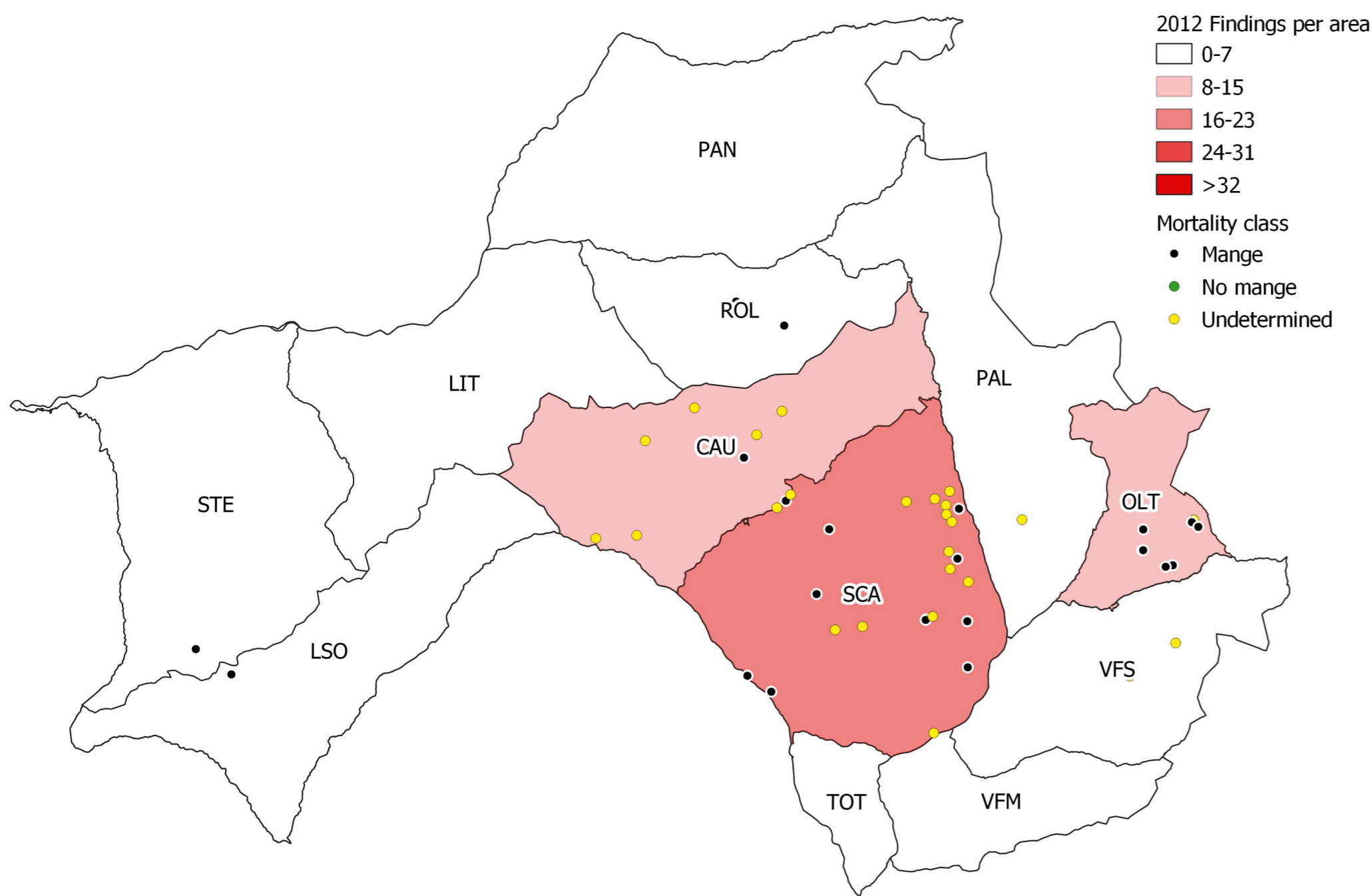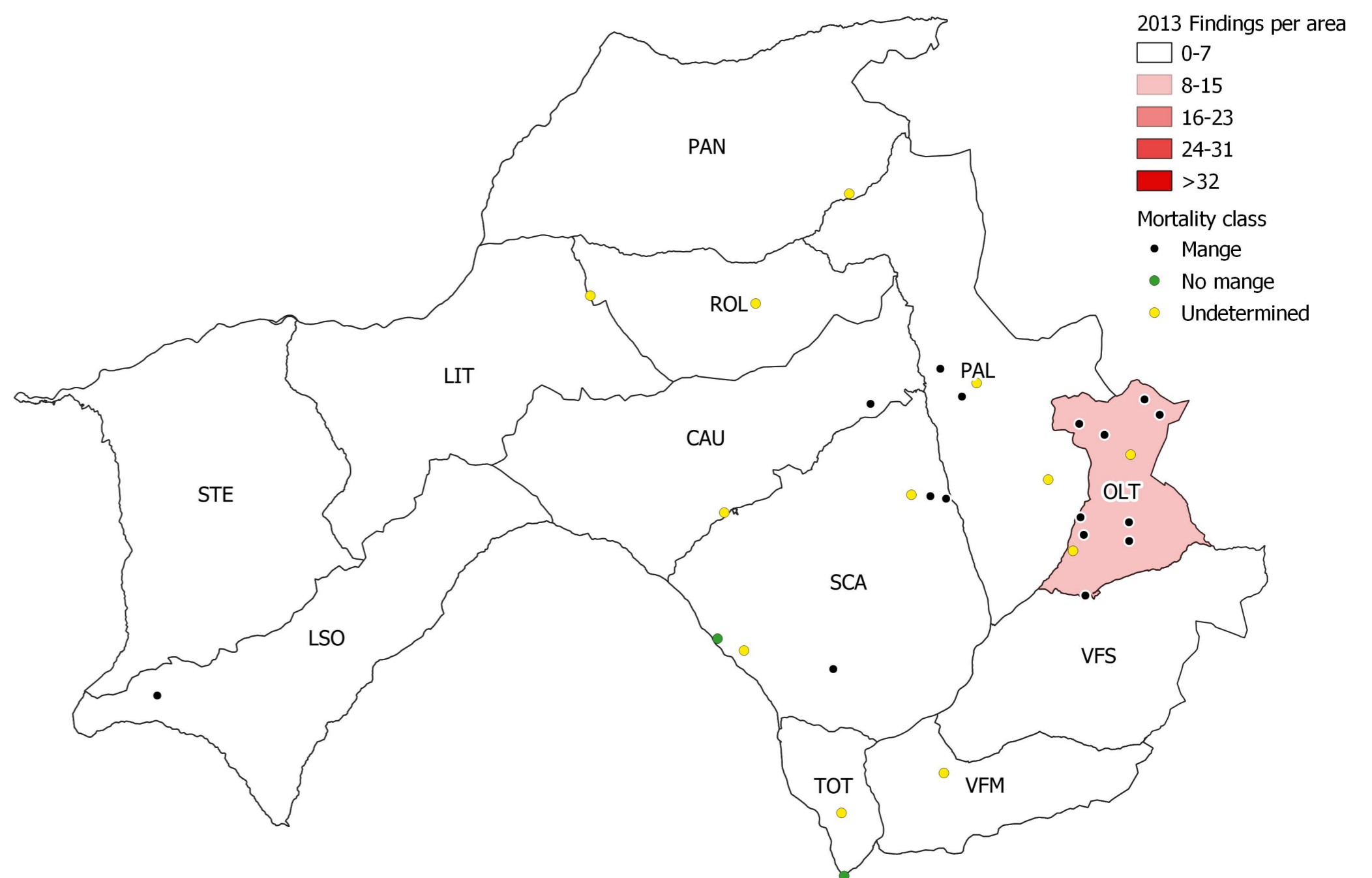

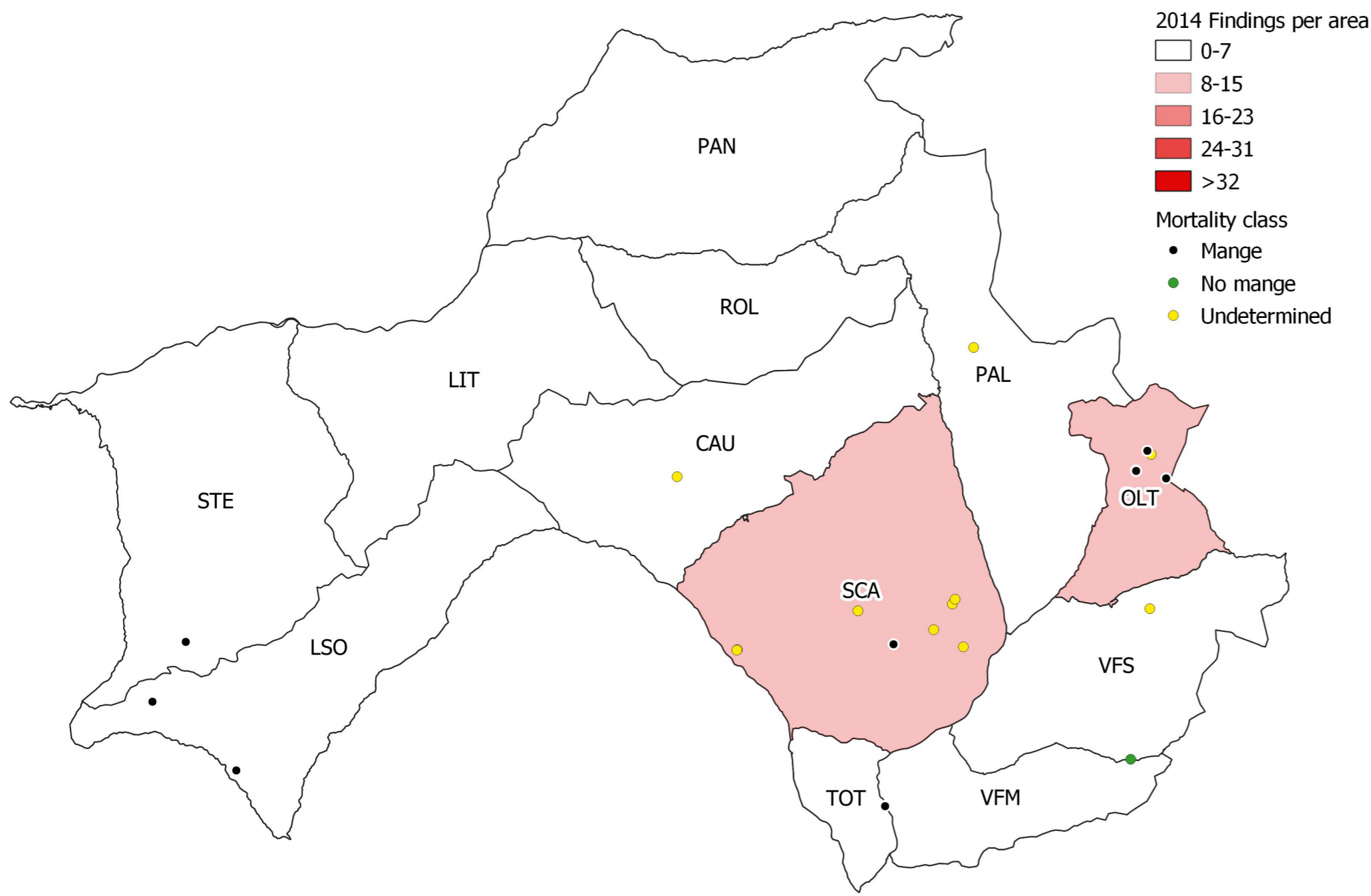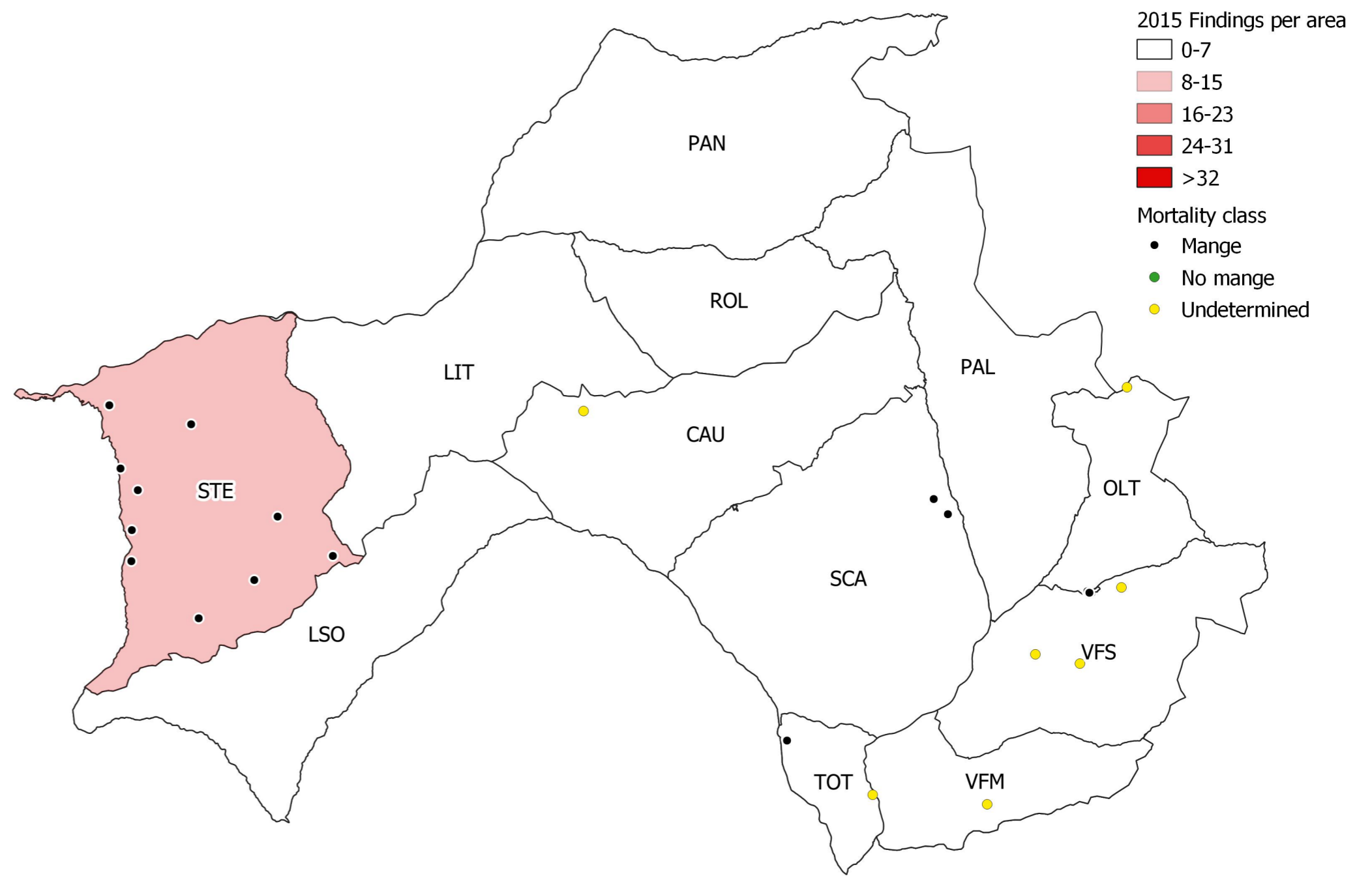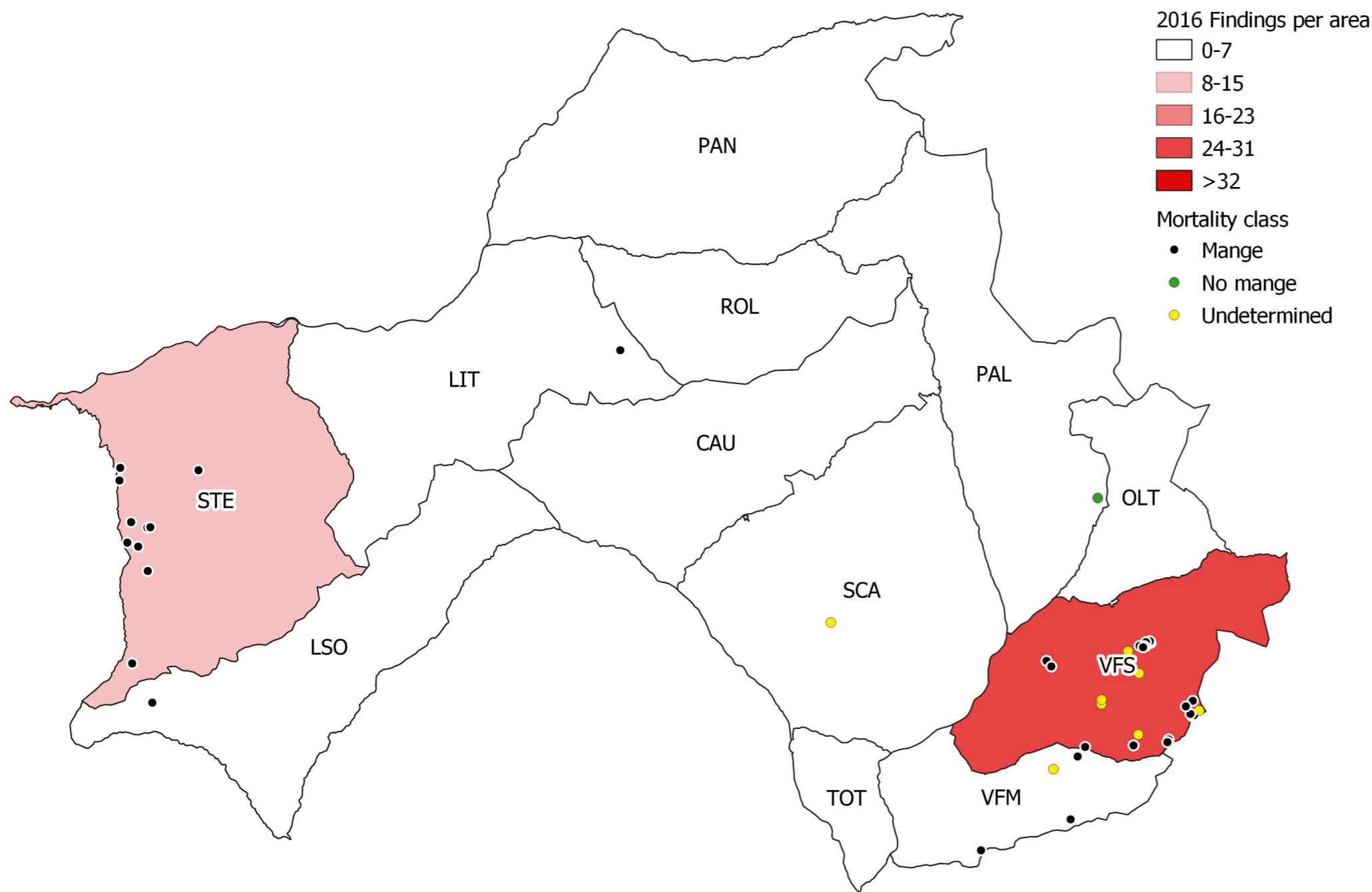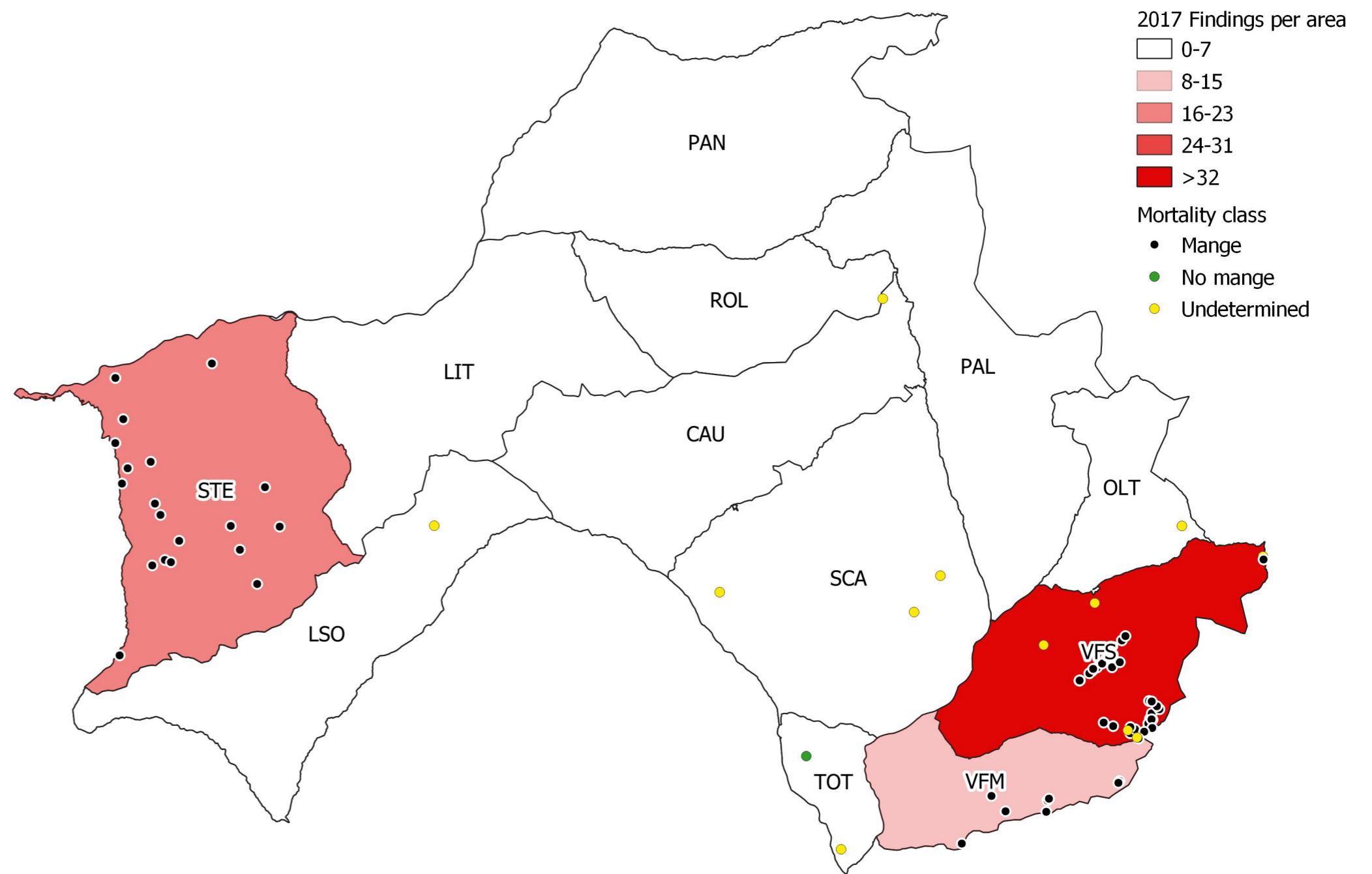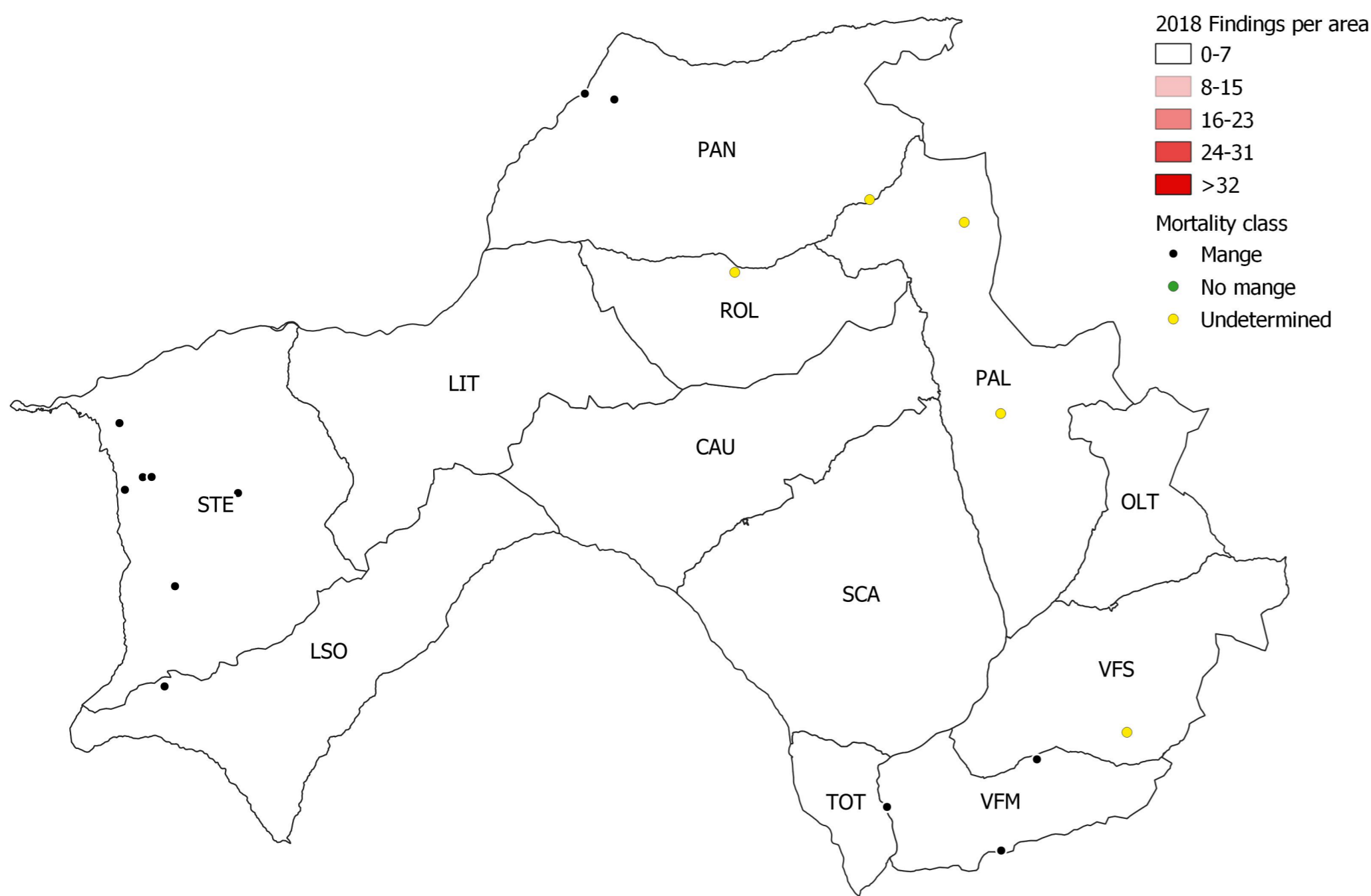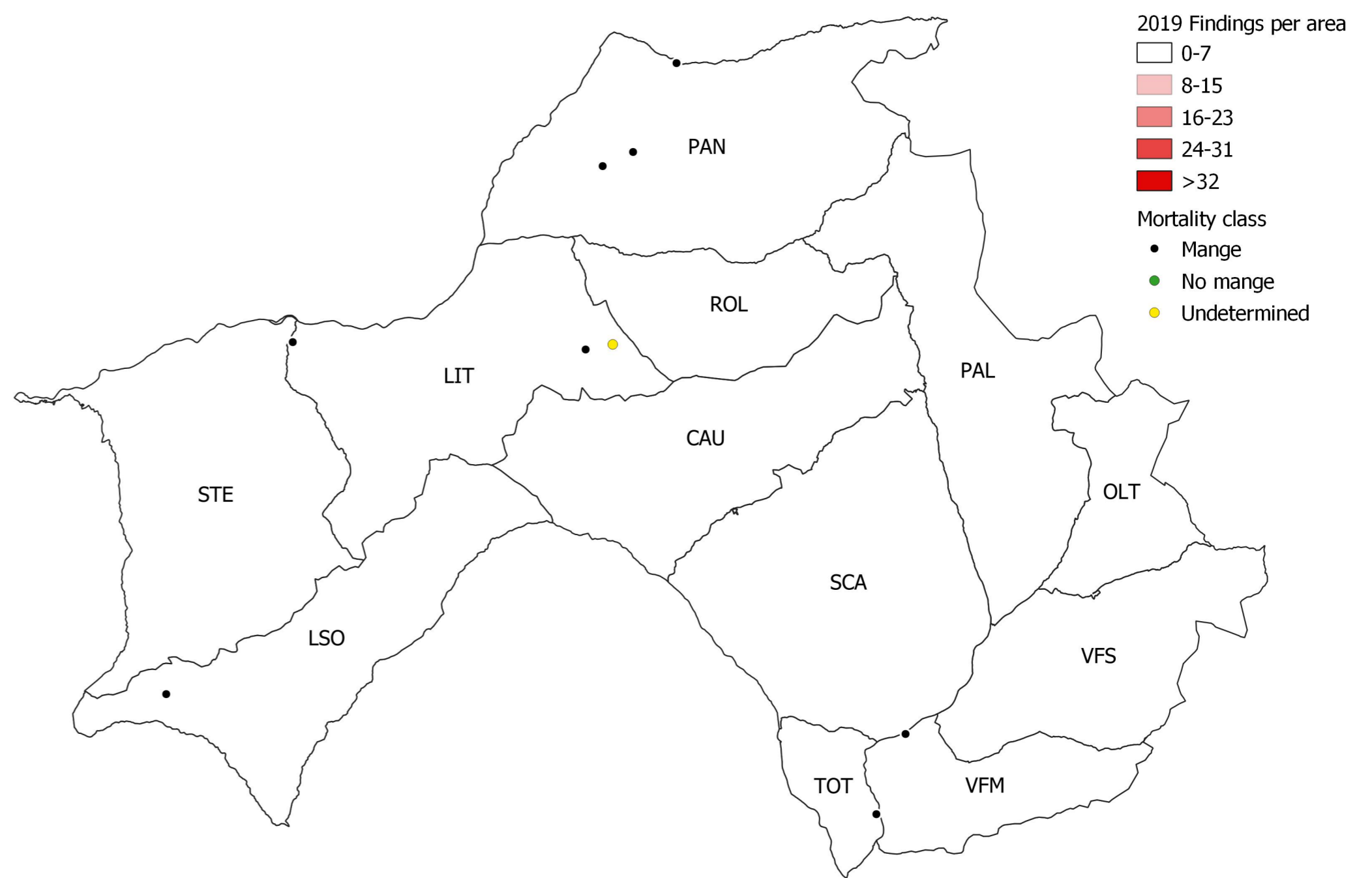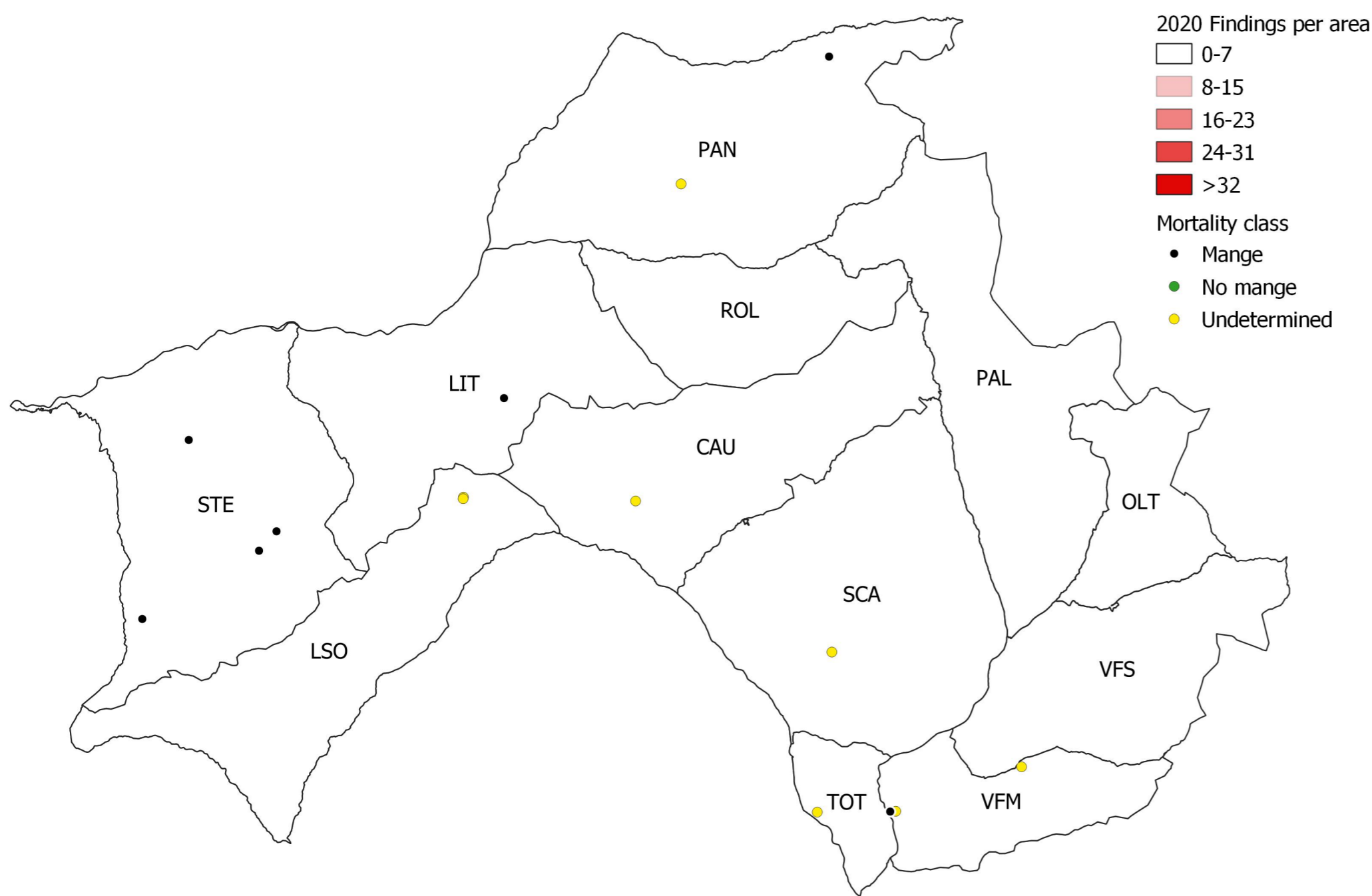

Supplement: Supplementary file 1 [file animals-12-02077-s001.zip › Figure S2_Surveillance maps.pdf]
